# Enhancing c-Si Solar Cell Efficiency in the UV Region: Photophysical Insights into the Use of Eu^3+^ Complexes for Down-Shifting Layer Applications

**DOI:** 10.3390/molecules28237924

**Published:** 2023-12-04

**Authors:** Fabian Vargas, Ronald Nelson, Dario Espinoza, Ivan Brito, Laura Sánchez-Muñoz, Pere Alemany, Sergio Ortiz, Pablo Ferrada, Alifhers Mestra, Jaime Llanos

**Affiliations:** 1 Departamento de Química, Universidad Católica del Norte, Avda. Angamos 0610, Antofagasta 1270709, Chile; fabian.vargas@alumnos.ucn.cl (F.V.); rnelson@ucn.cl (R.N.); despinoza01@ucn.cl (D.E.); alifhers.mestra@ucn.cl (A.M.); 2 Departamento de Química, Universidad de Antofagasta, Antofagasta 1240000, Chile; ibrito@uantof.cl; 3 Departamento de Ciència de Materials i Química Física, Institut de Química Teòrica i Computacional (IQTCUB), Universitat de Barcelona, Diagonal 647, 08028 Barcelona, Spain; sanchezlaura124@gmail.com (L.S.-M.); p.alemany@ub.edu (P.A.); 4 UMR 7200 Laboratoire d’Innovation Thérapeutique, CNRS, Strasbourg Drug Discovery and Development Institute (IMS), Université de Strasbourg, 67400 Illkirch-Graffenstaden, France; ortizaguirre@unistra.fr; 5 Centro de Desarrollo Energético Antofagasta, Universidad de Antofagasta, Angamos 601, Antofagasta 1240000, Chile; pablo.ferrada.m@uantof.cl; 6 Centro Lithium I+D+i, Universidad Católica del Norte, Avenida Angamos 0610, Antofagasta 1270709, Chile

**Keywords:** europium complex, c-Si solar cell, luminescent down-shifting layer, synthesis and characterization methods for optoelectronic materials

## Abstract

[Eu(^3^DPIQC)_3_] (where DPIQC = 3-(diphenyl phosphoryl)-1-isoquinolinecarboxylate), a luminescent europium complex with antenna ligands, has been carefully embedded within a polyvinyl butyral (PVB) matrix and the resulting material was used to prepare films used as luminescent down-shifting layers (LDSLs) for crystalline Si-based solar cells. The films were characterized using photoluminescence spectroscopy, atomic force spectroscopy (AFM), UV-Vis spectroscopy, and fluorescence microscopy. The AFM analysis shows films with low surface roughness, while fluorescence microscopy revealed that the Eu complex embedded in PVB assumed a spheroidal configuration, a morphology especially beneficial for optical applications. The so-obtained LDSLs were utilized as energy converters in c-Si solar cells to enhance the utilization of high-energy photons, thereby improving their overall efficiency. The determination of photovoltaic parameters carried out before and after the deposition of the LDSLs on the c-Si cells confirms a positive effect on the efficiency of the cell. The *J_sc_* increases from 121.6 mA/cm^2^ to 124.9 mA/cm^2^, and the open circuit voltage (*V_oc_*) is found to be unrelated to the complex concentration in the films. The fill factor (*FF*) remains constant with the Eu concentration. The EQE curves indicate an enhancement in the performance of the photovoltaic cells within the UV region of the spectrum for all coated devices. Electrochemical impedance spectroscopy (EIS) was also carried out in order to analyze the effect of the Eu complex in the charge transfer process of the devices.

## 1. Introduction

Rare-earth-containing compounds have been the subject of extensive research in recent decades, mainly due to their interesting photophysical properties such as high quantum yields, narrow emission lines, and long-term fluorescence [1,2,3,4]. Due to these properties, these compounds have found a great number of applications in the field of opto-electronics, such as in solid-state lighting devices, temperature sensors, displays, bio-imaging applications, or solar cells [5,6,7,8,9]. It is well known that due to the nature of the f-f transitions, the lanthanide cations present low absorption coefficients in the UV–visible regions. This inconvenience can, however, be easily circumvented in practical applications by using lanthanide complexes including ligands that act as antennas or sensitizers. Antenna ligands, with much higher absorption coefficients in the UV–visible range, are used to absorb and subsequently transfer energy to the central lanthanide cation, which is responsible for the final emission. This suggests that, besides the excitation energy of the ligand’s singlet state, the ligand’s triplet state, which can be directly generated by carrier recombination, can also be utilized to excite the emitter center. Consequently, the energy transfer efficiency can be optimized through the appropriate selection of antenna ligands, particularly by aligning the energy level of their triplet state with the excited states of the 4f ion of interest [10,11,12]. 

For some applications, specifically for enhancing the efficiency of solar cells, solutions of luminescent lanthanide complexes with very high quantum yields are introduced in transparent polymers with the objective of obtaining transparent thin films called luminescent down-shifting layers (LDSLs) that are incorporated into the solar cell to absorb high-energy radiation that would otherwise lead to thermal losses and convert it into low energy photons with wavelengths in the optimal absorption region of the photovoltaic material. An efficient LDSL should absorb ultraviolet and visible radiation, typically between 300 and 500 nm, and must re-emit at a wavelength where the external quantum efficiency (EQE) of the cell is near 100% (from 500 to 700 nm). These re-emitted photons will be efficiently absorbed by the PV cell, generating a large number of electron-hole pairs and thereby increasing its short circuit current (*J_sc_*) [13,14,15,16,17,18,19]. Common polymers for obtaining highly transparent thin films are poly (methyl methacrylate) (PMMA) and polyvinyl butyral (PVB) [20,21,22,23]. 

UV radiation is a well-known factor that impacts the performance of c-Si solar cells, primarily due to aging processes resulting from the generation of surface defects. To address these challenges and enhance the durability of minority carriers, the common practice is to apply dielectric layers like hydrogenated silicon nitride (SixNy:H) and silicon dioxide (SiO_2_) to passivate the solar cell surface. However, exposure to UV radiation can disrupt this passivation process by damaging either the passivation layer itself or the interface between the passivation layer and the silicon cell. Additionally, it can result in subsurface damage to the silicon material. Various research groups have identified specific wavelengths within the 250–400 nm spectral range to be responsible for the UV-induced degradation observed in silicon solar cells [24,25,26]. We propose the use of down-shifting luminescent layer coating not only as a means to improve the cell’s efficiency through harvesting photons in the UV range, but also as a viable alternative to avoid the degradation of the cells caused by intense UV exposure. The integration of down-shifting layers in conjunction with luminescent solar concentrators (LSCs) can yield a substantial enhancement of approximately 20% in the solar cell’s overall efficiency [27]. 

Recently, Cai et al. reported on the synthesis and photophysical properties of an Eu (III) complex based on 3-(diphenylphosphoryl)-1-isoquinolinecarboxilic acid (H^3^DPIQC) [28]. This ligand, with an enlarged π conjugation, was found to act as an efficient antenna with a strong absorption in the 240–350 nm region. Upon excitation with 330 nm radiation, the resulting Eu^3+^ complex, Eu(^3^DPIQC)_3_, showed an intense red emission with a very high quantum yield in solution as well as in solid-state, 0.69 and 0.94, respectively. These features make this Eu (III) complex an ideal candidate for LDSLs in silicon-based photovoltaic devices. To the best of our knowledge, a luminescent down-shifting layer containing Eu(^3^DPIQC)_3_ has not been reported for c-Si solar cells, and the purpose of this paper is the fabrication, characterization, and determination of the optical properties of LDSLs containing Eu(^3^DPIQC)_3_ embedded in polyvinyl butyral (PVB). The characterization includes the determination of their thermal stability, XPS studies, and the recording of excitation and photoluminescent emission spectra. In order to gain more information on the complete photophysical process, INDO/S-CIS calculations were performed to obtain the energy diagram for Eu(^3^DPIQC)_3_, the character of the S_1_ and T_1_ excited states, and the concurrent ligand → Eu (III) energy transfer. In addition, the doped polymer films were spin-coated on the top of an n-type passivated emitter rear totally diffused (n-PERT) solar cell and the I-V curves and external quantum efficiency (EQE) were measured in order to demonstrate that the incorporation of the LDSL containing the Eu (III) complex effectively enhances light harvesting in the ultraviolet range of the solar spectrum.

## 2. Results and Discussion

### 2.1. Synthesis and Characterization

**Scheme 1:**
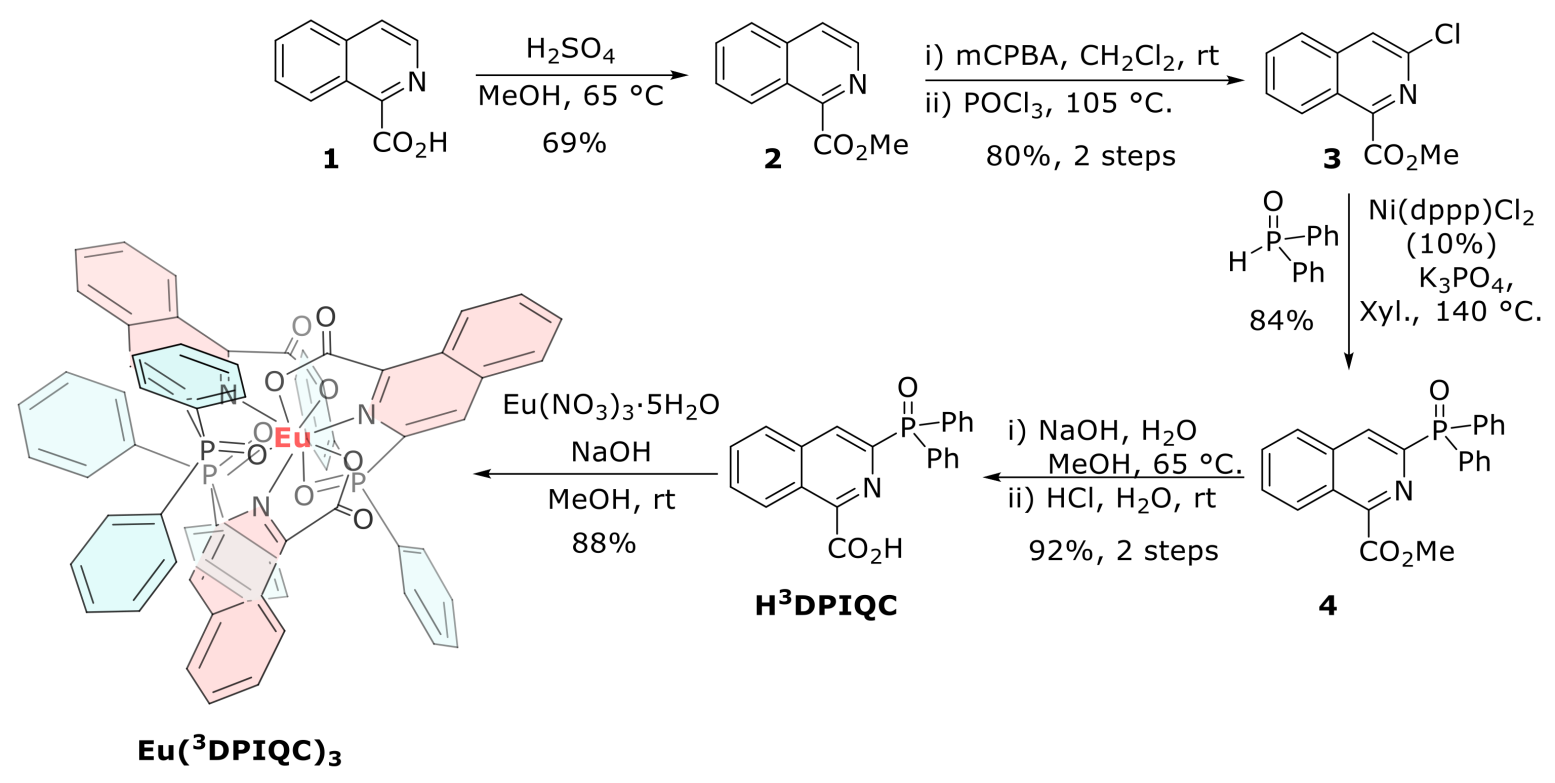
Synthesis of the Eu(^3^DPIQC)_3_ complex.

Eu(^3^DPIQC)_3_ was synthetized following the procedure described in ref. [28]. The synthesis began with the esterification of 1-isoquinolinecarboxylic acid (**1**), followed by *N*-oxidation and chlorination with POCl_3_, obtaining chloroisoquinoline **3** in a 55% yield (three steps). The next step, a C-P cross coupling, gave low yields with the originally described procedure between compound **3** and diphenylphosphine oxide (DPPO). For this reason, it was necessary to perform an optimization of the nickel-catalyzed procedure (see Table S1 in Supplementary Materials), finding that the best conditions to obtain isoquinoline **4** were using Ni(dppp)Cl_2_ (10 mol%) as catalyst and K_3_PO_4_ as the base in xylene at 140 °C (under argon atmosphere), allowing the desired product to be obtained at an 84% yield on a scale of up to 4.0 mmol. The tridentate ligand H^3^DPIQC was obtained via the hydrolysis of the methyl ester using NaOH in MeOH, followed by neutralization with HCl. Finally, the Eu(^3^DPIQC)_3_ complex was prepared by a deprotonation of the ligand with NaOH and one third equivalent of Eu(NO_3_)_3_·5H_2_O in MeOH, achieving a 88% yield. The synthesis steps are detailed in Scheme 1.

**Figure 1:**
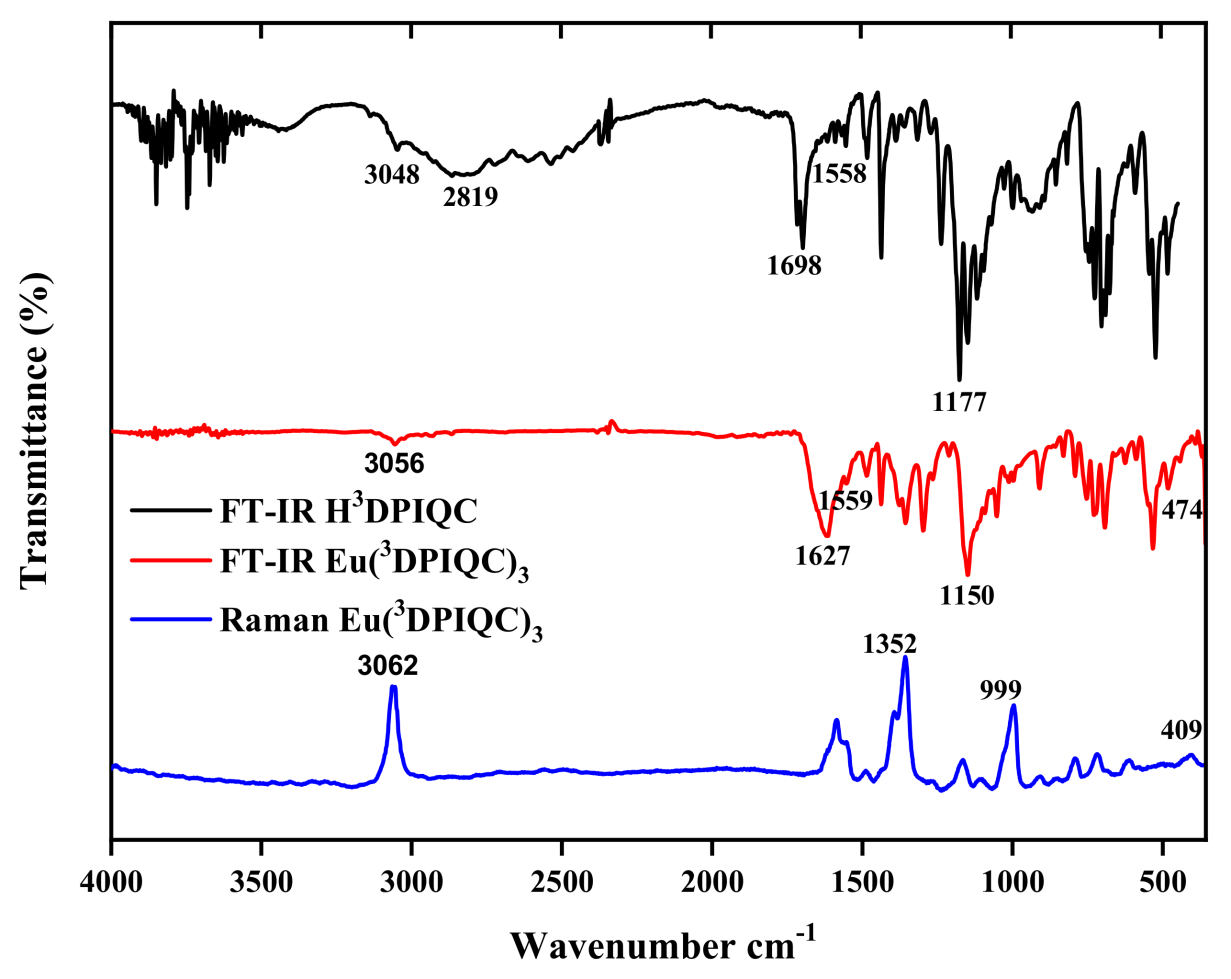
FT-IR spectra of the H^3^DPIQC ligand (black) and Eu(^3^DPIQC)_3_ complex (red) and Raman of the Eu(^3^DPIQC)_3_ complex (blue).

All synthetic intermediates were structurally characterized by FT-IR, ^1^H-NMR, ^13^C-NMR, ^31^P-NMR, and HRMS-ESI. The structure of the Eu(^3^DPIQC)_3_ complex was also verified by comparing the X-ray powder diffraction pattern with the calculated pattern for the single crystal structure (see Figure S1) [28]. The FT-IR and Raman spectra of the Eu(^3^DPIQC)_3_ complex and the H^3^DPIQC ligand are shown in Figure 1. The bands observed at 1627 and 1549 cm^−1^ in the Eu complex are assigned to C=O and C=N stretching vibrations of the ligand [29,30]. These bands exhibit frequency shifts towards higher values compared to those of the corresponding free ligand. (1698 and 1558 cm^−1^, respectively). The notable shift of the C=O band between H^3^DPIQC and the Eu complex, coupled with the absence of the broad band centered at 2819 cm^−1^ in the ligand’s spectrum, provides compelling evidence for the coordination of the carboxylate group with the Eu^3+^ ion [31]. The IR spectrum of the Eu complex also shows a strong band at 1150 cm^−1^ that can be attributed to the P=O stretching vibrational modes of the phosphoryl moiety [29,32], whose frequency is also shifted to higher frequencies than in the corresponding free ligand (H^3^DPIQC). The band linked to Eu-O was observed at 474 cm^−1^, while Eu-N vibration was not observed in FT-IR. However, the Eu-N band was observed at 409 cm^−1^ in the Raman spectrum [33,34]. Additionally, the formation of the Eu(^3^DPIQC)_3_ complex could be confirmed by HRMS-ESI analysis, which shows a molecular ion [M + Na]^+^ of *m*/*z* = 1290.1423, corresponding to a molecular formula C_66_H_45_O_9_N_3_P_3_EuNa.

### 2.2. Optical Properties of the Eu(^3^DPIQC)_3_ Complex in Solution and in PVB Films

The optical properties of Eu(^3^DPIQC)_3_ in solution have already been reported by Cai et al. [28]. In order to gain some deeper knowledge on the origin of the bands observed in the absorption spectrum, we have computed them with LUMPAC [35]. For this, we have first optimized the structure of the complex using the RM1/Sparkle model [36]. The optimized structure is in close agreement with the X-ray structure reported by Cai et al. [28]. The europium atom is placed in a nine-fold coordination environment formed by six oxygen and three nitrogen atoms. According to continuous shape measures, the closest coordination polyhedron is the Johnson tricapped trigonal prism (J51 solid) with 14 equilateral triangles as its faces [37,38]. The shape measurements for the coordination polyhedron in the crystal structure and in the optimized geometry, 1.862 and 2.054, respectively, indicate a moderate distortion compared to the ideal geometry. Continuous symmetry measures show that the C_3_ axis is preserved both in the crystal and the optimized structure, with S (C_3_ ≈ 0). Due to this C_3_ symmetry, the coordination environment of Eu is far from being centrosymmetric, with S(C_i_) = 14.68 and 14.63 for the crystal and optimized structures, respectively. These large values of S(C_i_) are requisite for intense emission from the f-f transitions, which are strictly prohibited in a perfectly centrosymmetric environment with S(C_i_) = 0. 

**Figure 2:**
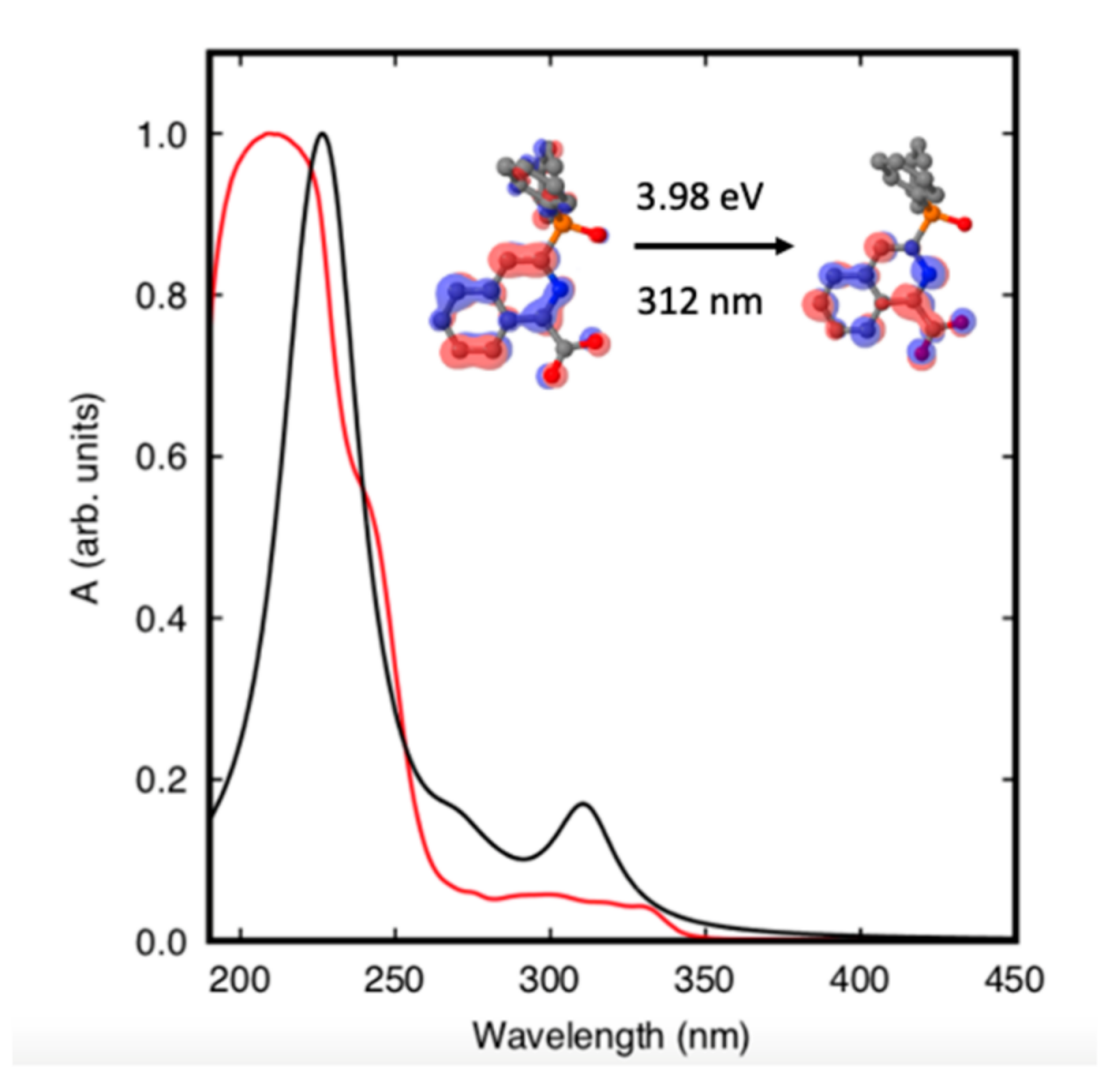
Experimental (red line) and calculated (black line) absorption spectra of Eu(^3^DPIQC)_3_ in solution (1 × 10^−5^ M in CH_2_Cl_2_). The inset shows the ligand’s molecular orbitals involved in the transition to the lowest excited singlet state.

**Figure 3:**
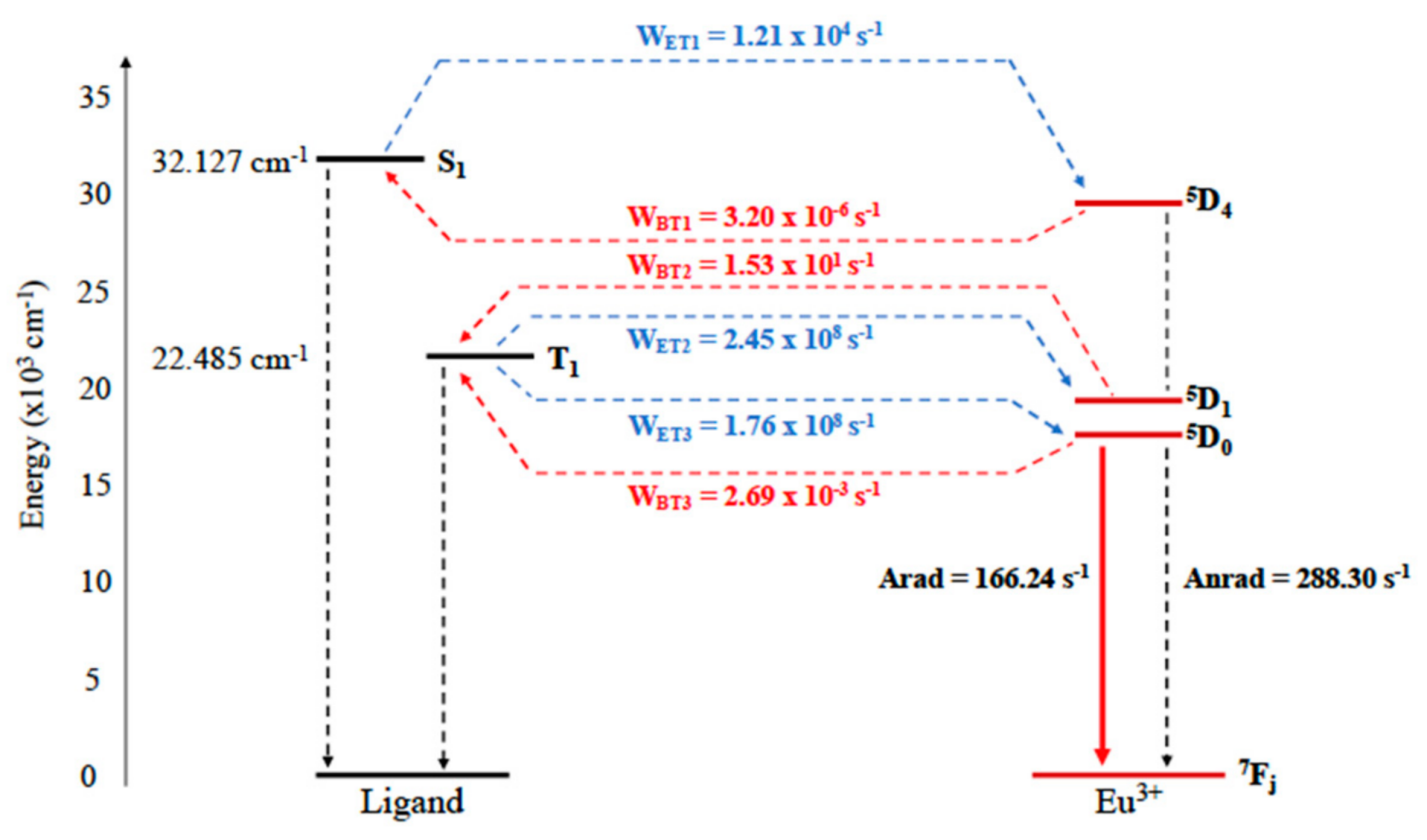
Calculated Jablonsky diagram for Eu(^3^DPIQC)_3_, indicating the transition probabilities for energy transfer between the ligands and the central Eu^3+^ cation (blue dashed arrows) and the corresponding backward transfers (red dashed arrows).

The calculated UV-vis spectrum (Figure 2) coincides well with that reported by Cai et al. [28]. The peak at 312 nm in the computed spectrum can be attributed to a π → π* transition in one of the three ^3^DPIQC ligands (see inset in Figure 2), leading to the lowest excited singlet state S_1_, which appears at 3.98 eV (32,128 cm^−1^) above the ground state. The ^3^DPIQC ligand is a triplet sensitive ligand so that efficient transfer from S_1_ to the lowest triplet state T_1_, calculated to lie 2.79 eV (22,485 cm^−1^) above the ground state, is expected [10,39]. As shown in Figure 3, the T_1_ state is well aligned for an effective energy transfer between the ligands and the ^5^D_1_ and ^5^D_0_ levels of the central Eu^3+^ cation.

The energy transfer rates from the singlet and triplet excited states to the Eu^3+^ cation (Figure 3) show, indeed, that transfer from the ligands to the central atom is dominated by the T_1_ to ^5^D_1_ and ^5^D_0_ transitions, while back transfer is practically irrelevant in this case. The model included in LUMPAC to estimate energy transfer rates is able to predict that the Eu(^3^DPIQC)_3_ complex is a highly effective down-shifting material, although the actual predicted quantum yield, 36.2%, is lower than the 69% value reported by Cai et al. [28]. This discrepancy is, however, within the bounds of what can be considered to be acceptable, taking into account that predicting the quantum yield with high accuracy is a difficult task. Since several mechanisms (radiative and non-radiative deactivation of the ligand through different channels, intraligand transfer from S_1_ to T_1_, transfers from S_1_ and T_1_ to the ^5^D_J_ levels of Eu^3+^ with all corresponding back-transfers, and radiative and nonradiative deactivation of the Eu^3+^ center) operate simultaneously, an accumulation of errors in the estimation of the rate for each channel can yield a substantial error in the final value for the quantum yield.

**Figure 4:**
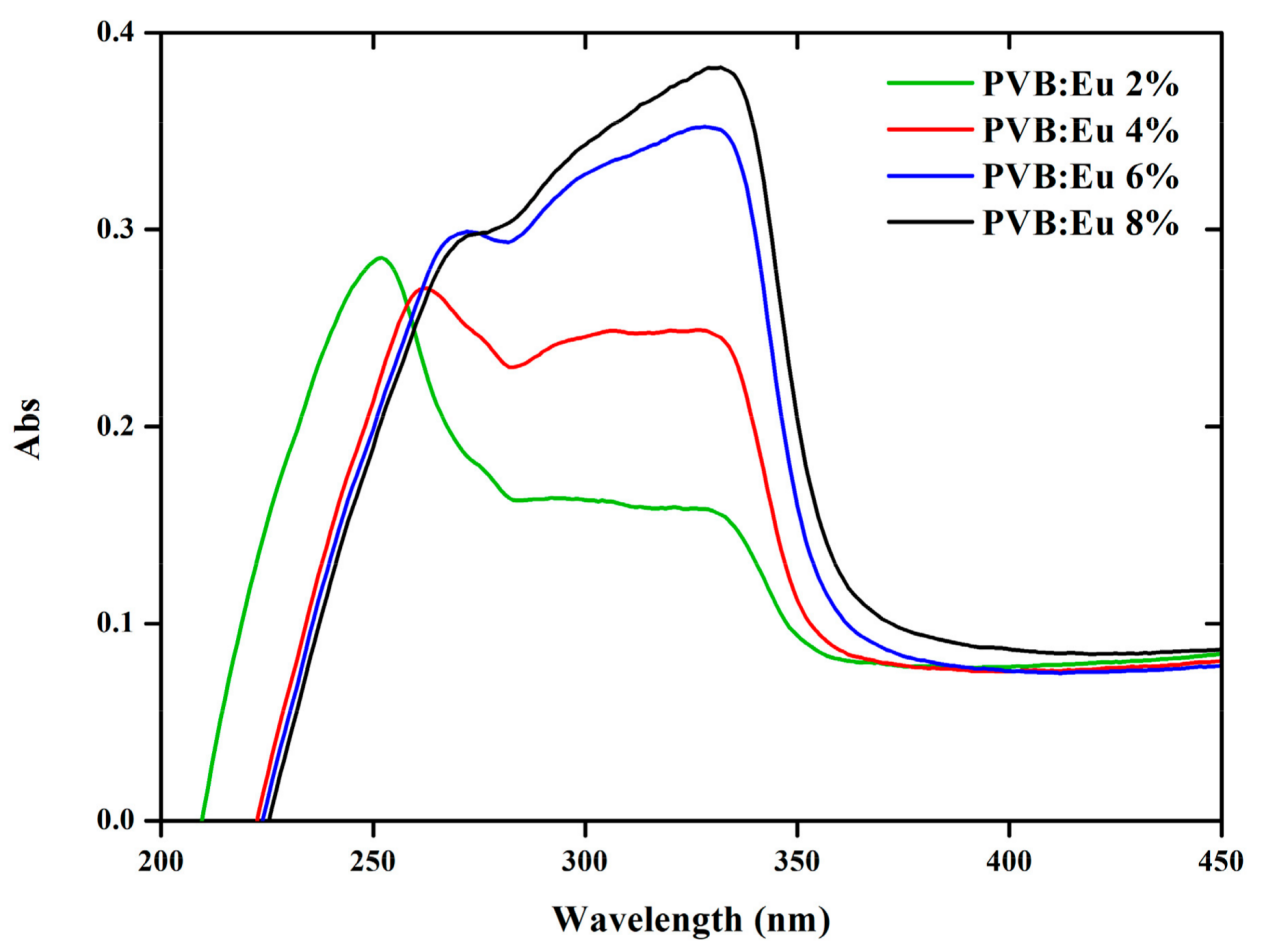
Absorption spectra of PVB (polyvinyl butyral) films with varying proportions of Eu(^3^DPIQC)_3_.

For our research, we incorporated the Eu(^3^DPIQC)_3_ complex into polymer matrices, and subsequently LDSL films were coated onto glass substrates using the spin-coating method. Figure 4 shows the absorption spectra of PVB (Polyvinyl butyral) films with varying proportions of Eu(^3^DPIQC)_3_, namely 2%, 4%, 6%, and 8%. In all cases, an absorption peak at approximately 345 nm was observed. It is evident that as the concentration of Eu within the PVB matrix increased, there was a noticeable redshift in the absorption band. This bathochromic shift is associated with an increase in the electron density surrounding the Eu(III) ion, given that the polymeric matrix can be viewed as a low-polarity solvent [40].

**Figure 5:**
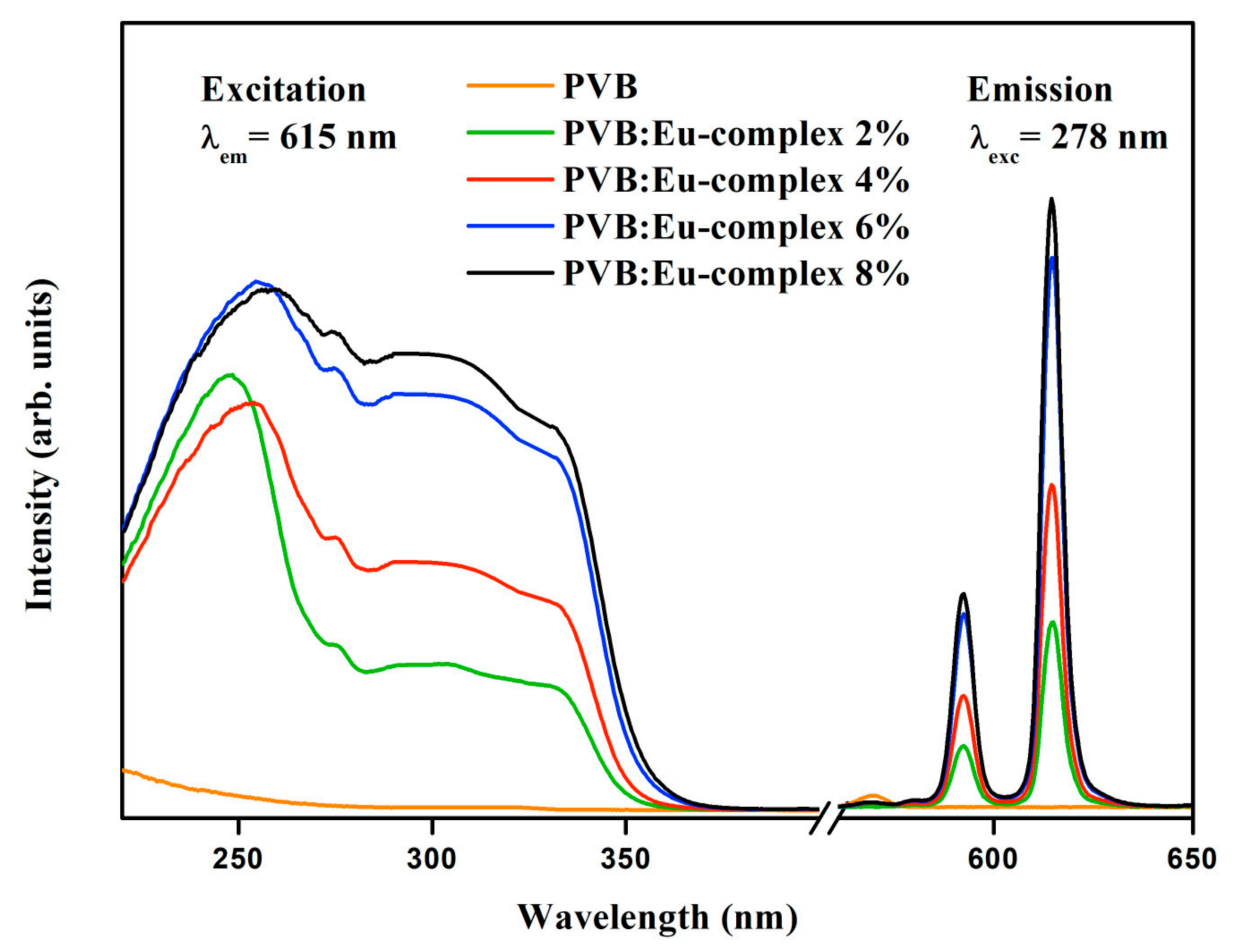
Excitation (λ_em_ = 615 nm) and emission spectra (λ_ex_ = 278 nm) for the ^5^D_0_ → ^5^D_j_ transition in Eu complex with different proportions of Eu^3+^ embedded in the PVB matrix.

The fluorescence excitation and emission spectra of the Eu(^3^DPIQC)_3_ complex in solution (1 × 10^−5^ M in CH_2_Cl_2_) were previously investigated and reported on by Cai et al. [28]. The excitation spectrum exhibited a broad band with an edge at approximately 350 nm, while the emission spectrum displayed distinct peaks at 580, 590, 615, and 700 nm. The emission spectra for PVB films with different concentrations of Eu complex are shown in Figure 5. All samples were excited at 278 nm, resulting in the observation of the ^5^D_0_ → ^7^F_1_ transition at 590 nm, and the most intense peak, corresponding to the red ^5^D_0_ → ^7^F_2_ transition, at 615 nm. Some Eu^3+^ transitions exhibited a remarkable sensitivity to the coordination environment. Specifically, the ^5^D_0_ → ^7^F_1_ transition is classified as a magnetic dipole (MD) transition, and interestingly, it appears to be largely unaffected by the chemical environment surrounding the Eu^3+^ cation, whereas the ^5^D_0_ → ^7^F_2_ transition is an electric dipole (ED) transition and is often referred to as a “hypersensitive transition”. This term implies that its occurrence and intensity are significantly influenced by the local symmetry of the Eu^3+^ ion [41,42].

The emission spectra of the Eu complex do not show any visible transitions originating from the ligand. This observation suggests that the energy absorbed by the ligand is not directly emitted by the ligand itself. Instead, the absorbed energy is transferred to the Eu^3+^ cation. According to Zhang, the integrated intensity ratio of ^5^D_0_→^7^F_2_ to ^5^D_0_ → ^7^F_1_ transitions can serve as a useful indicator for evaluating the symmetry and monochromaticity of the coordination sphere in a lanthanide complex [43].

By examining this intensity ratio, it is possible to gain insights into the local environment around the lanthanide ion and determine the degree of symmetry present in the complex. The evaluation of this ratio in the samples excited at 278 nm gives 2.4 ± 0.2 for the complex in solid state, whereas for Eu(^3^DPIQC)_3_ PVB films the values range from 2.9 to 2.8 ± 0.2, which indicates that when the Eu complex is embedded in PVB a slight reduction in symmetry around the Eu^3+^ ion is observed. Consequently, one can deduce a slight decrease in the intensity of the ^5^D_0_ → ^7^F_2_ hypersensitive electric dipole transition of the ion Eu^3+^.

The calculated Judd–Ofelt parameters for Eu(^3^DPIQC)_3_ are shown in Table 1, together with those obtained from experimental emission spectra, both in solution and in PVB films with different concentrations of Eu(^3^DPIQC)_3_. While all three intensity parameters can be obtained in the theoretical calculation, the Ω_2_ and Ω_4_ parameters are the only ones achievable from luminescence spectra as the ^5^D_0_ → ^7^F_6_ transition remains unknown and the determination of Ω_6_ is impeded by the lack of dipole strength information for this transition. The experimental and calculated Ω_2_ and Ω_4_ values for the Eu complex in solution are in good agreement and similar to those obtained for the compound in PVB:Eu films with different concentrations of the active complex. If we disregard Ω_6_, which is found from the calculation to be much smaller than the other two intensity parameters, we can observe that Ω_2_ steadily increases with concentration in the polymer films, suggesting that the PVB matrix influences the chemical environment of the Eu^3+^ ion, inducing a small increase in the covalency in the first coordination sphere Eu. On the other hand, the Ω_4_ parameter is also somewhat higher in the polymer films than for the pure complex. These changes may be interpreted as a weak perturbation introduced by the polymer matrix on the chelate effect of the tridentate ligands due to the steric effects [44,45]. According to Judd, large values of Ω_2_ can be expected for systems with a triangle as the coordination polyhedron, but small values of Ω_2_ are determinate for systems with a tricapped trigonal prism as a coordination polyhedron [46,47]. The Ω_2_ values determined in this work are in good agreement with a tricapped trigonal prism coordination around the Eu^3+^ ions suggested by Cai et al. [28] and found in the continuous shape measures analysis above. The radiative and non-radiative emission rates obtained from the Judd–Ofelt model predict a slight increase in both of them as the concentration of Eu(^3^DPIQC)_3_ in the PVB films increases.

### 2.3. Addition of an LDSL to a c-Si Solar Cell

**Figure 6:**
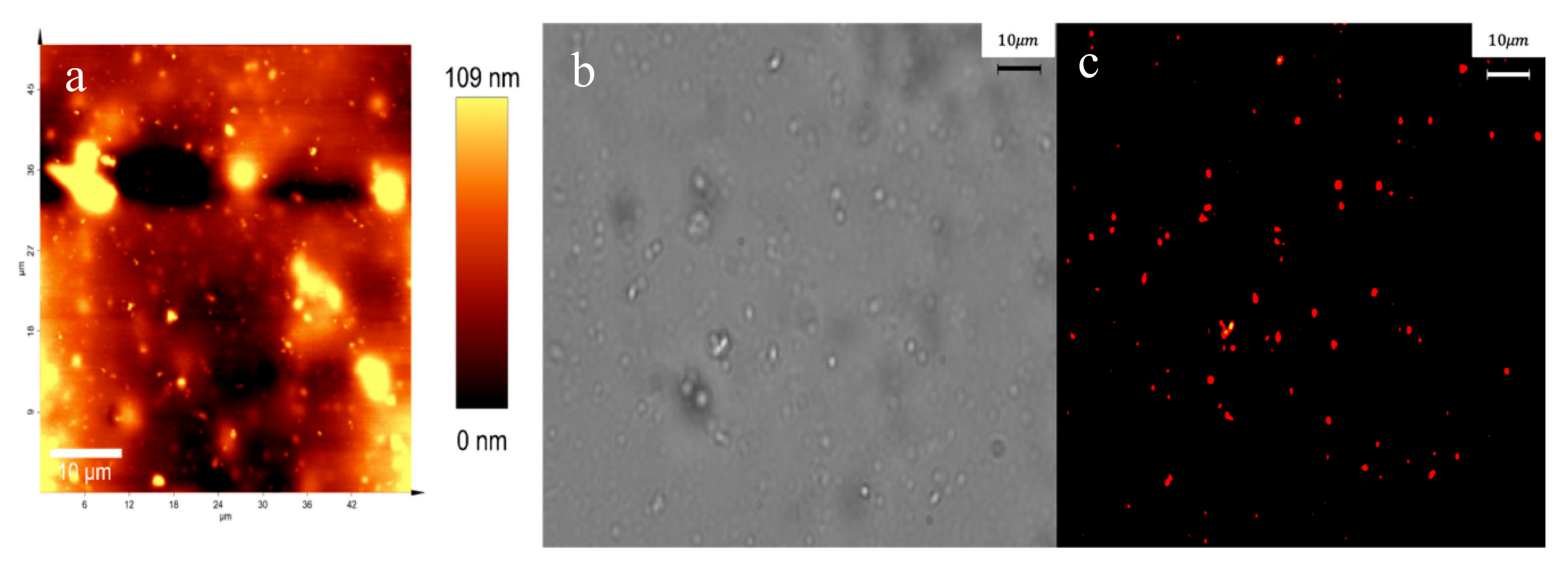
Two-dimensional AFM image of PVB: Eu6% (**a**). Dark field (**b**). Fluorescence microphotography of PVB:Eu6% (**c**).

An effective LDSL designed for photovoltaic applications should exhibit exceptional qualities such as significant light transmittance coupled with minimal light-scattering properties. The thickness and the roughness of the film are important parameters for such applications. The AFM analysis indicates that the PVB:Eu 6% films exhibit low surface roughness (see Figure 6a). On the other hand, the phenomenon of light scattering is especially applicable to spherical particles, as elucidated by the Mie theory [48]. In this investigation, the morphology of the PVB:Eu LDSLs was examined using fluorescence microscopy, revealing that the Eu complex embedded in PVB takes on a spheroidal shape. Figure 6 shows the two-dimensional surface (Figure 6a) as well as the dark field (Figure 6b) and fluorescence (Figure 6c) images of PBV:Eu 6% films. Based on these findings, it can be inferred that the presence of the matrix does not lead to a significant reduction in light absorption. Therefore, it is plausible to suggest that non-radiative deactivation might not play a significant role in this particular case, and the Eu complex embedded in PVB seems to be an efficient material to build LDSLs for improving the efficiency of c-Si solar cells.

**Figure 7:**
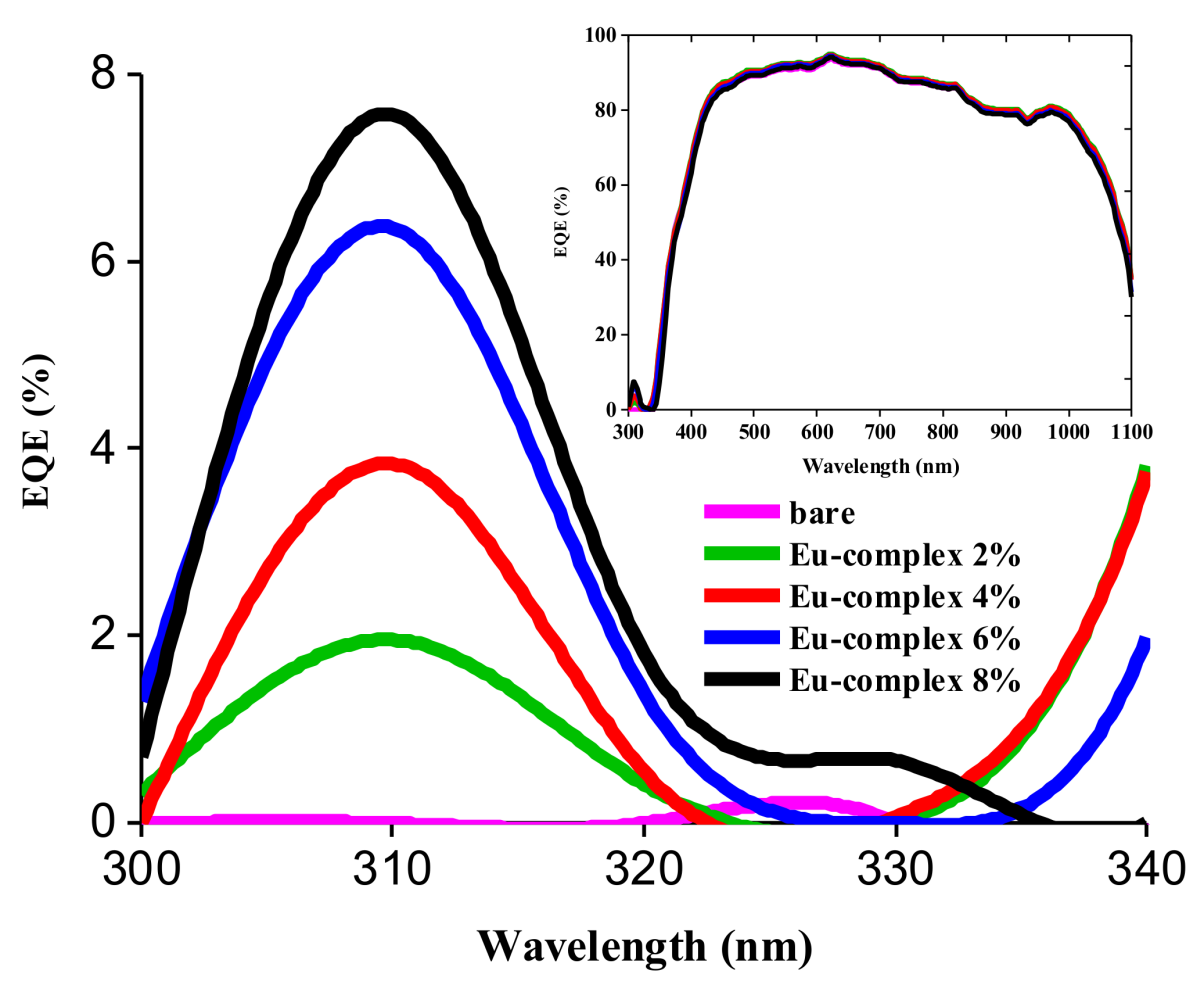
EQE curves in the UV region of a c-Si solar cell with an LDSL with different concentrations of Eu complex in PVB. The inset shows the EQE in the range between 300 and 1100 nm.

The effective contribution of the LDSL coating in enhancing the performance of the photovoltaic device was deduced through an analysis of the EQE curves for both the bare cell and the cells coated with PVB films containing different concentrations of the Eu complex. The EQE spectra of the photovoltaic device reveals a noticeable enhancement in the spectral response within the UV/blue region, specifically between 300 and 360 nm, when it is coated with an LDSL. This range is significant because c-Si cells typically experience reduced performance in this region due to cell overheating (see Figure 7).

The photocurrent density (*J_sc_*) of the PV device was determined by integrating the EQE in the 300–1100 nm range (see Figure 7). The relationship between the *J_sc_* and Eu complex concentration in PVB is shown in Table 2. It is possible to observe that the J*_sc_* value exhibits a slight variation up to the 3% concentration, followed by a slight increase at the 6% concentration. However, the photocurrent starts decreasing again as the concentration reaches 8% of the Eu complex. According to Regalado-Perez et al., this finding can be explained using the Lambert–Beer law [30]. As the concentration of Eu in the LDSL increases, a larger number of short wavelength photons are absorbed and converted into red photons, resulting in an increase of the photocurrent. While the absorbance is an exponential function of the concentration, the relationship between *J_sc_* and the Eu concentration shows a maximum at Eu:6%. This has been interpreted considering that a rise in Eu complex concentration within the PVB matrix will also obstruct the utilization of incident light on the cell, resulting in a decrease in the cell’s efficiency. The open circuit voltage (*V_oc_*) does not depend on the Eu complex concentration, whereas the fill-factor (FF) shows only a slight nonsystematic variation with the Eu concentration. Our results show that the *J_sc_* of the solar cell containing the LDSL is increased from 30.45 mA/cm^2^ for the bare cell to 31.10 mA/cm^2^ for the Eu:6% coated cell. 

In Table 2, the photovoltaic parameters of the c-Si solar cell are presented, showing their dependency on the concentration of the Eu complex embedded in PVB. Results reveal a 3% increase in cell efficiency when the cell is coated with 6% Eu complex. Conversely, the average external quantum efficiency (EQE) values, computed at three different wavelengths (300–330 nm, 330–400 nm, and 400–1000 nm), exhibit a satisfactory correspondence with the photoluminescent properties of Eu(^3^DPIQC)_3_.

The *J_sc_* values obtained from the EQE spectra were determined using the equation described by W.-J. Ho et al. [49]. The calculated results demonstrate a strong concordance with the experimental data, affirming that the coating with a 6% concentration of Eu complex is the most effective in enhancing the efficiency of the c-Si solar cell.

Our findings indicate that the utilization of Eu (III) 3-(diphenylphosphoryl)-1-isoquinolinecarboxylate is similar to other photoluminescent compounds embedded in different polymers. Table 3 shows the results reported for the EQE increase in the UV region of the spectra for some Eu^3+^-based LDS coatings.

**Figure 8:**
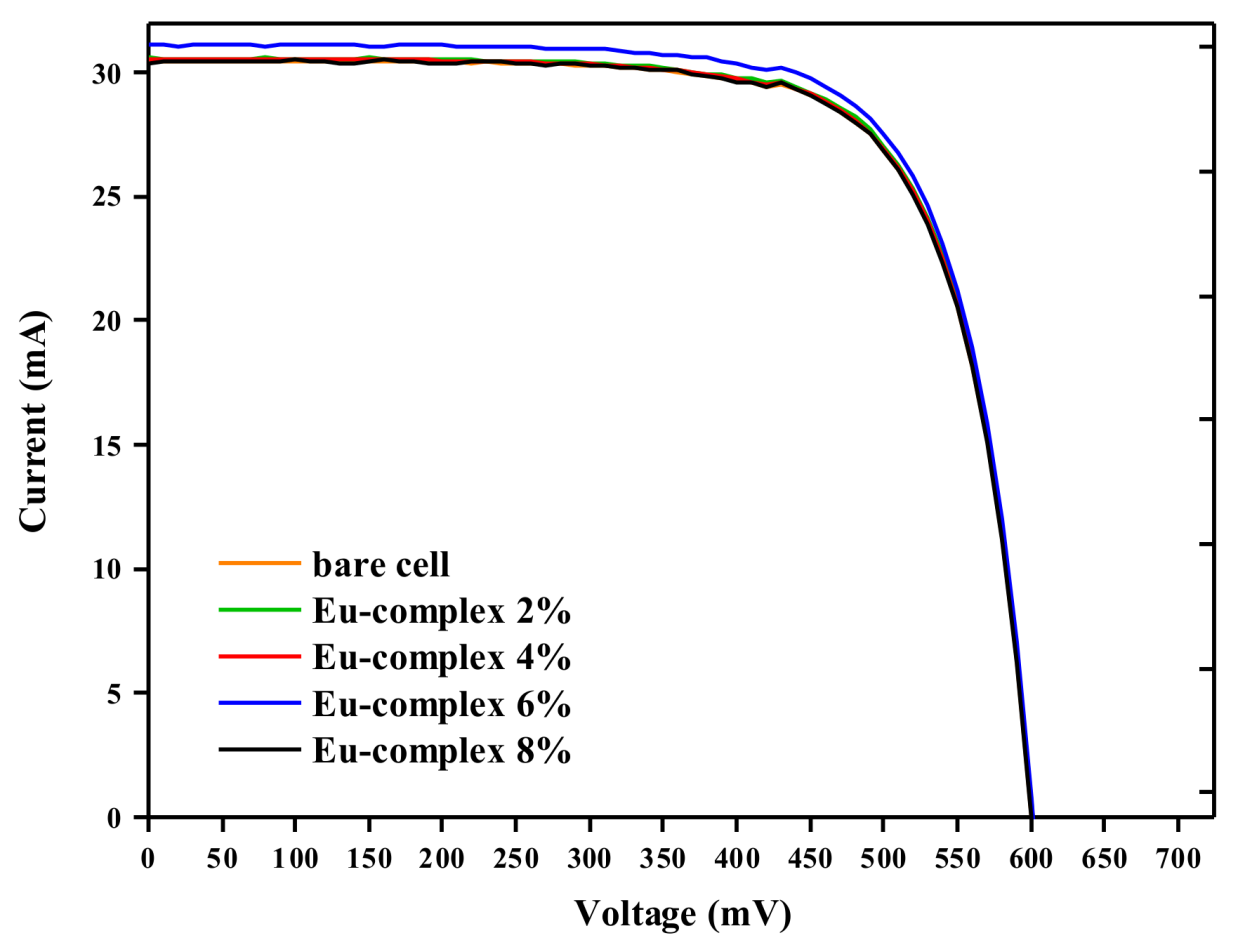
I–V curves obtained under the irradiation of standard AM1.5G of different concentrations of Eu(^3^DPIQC)_3_ complex and bare c-Si solar cell.

Consequently, the application of this complex as the active material in LDSLs leads to an increase in the efficiency of the c-Si solar cell. Detailed I–V curves are shown in Figure 8.

The enhanced electrical power generation from the photovoltaic device with the LDSL coating was deduced by the following equation [7]:

(1) $$\Delta PV=\frac{\int_{0}^{Voc}P_{coated}\;dV-\int_{0}^{Voc}P_{bared}\;dV}{\int_{0}^{Voc}P_{coated}\;dV}\times 100$$

where *V_oc_* represents the open-circuit voltage of the photovoltaic device, *P_coated_* the power generated by the LDSL-coated device, and *P_bare_* the power generated by the uncoated c-Si cell. The outcomes indicate an enhancement in cell efficiency, specifically a 2.3% increase for the cell coated with 6% Eu complex.

### 2.4. Electrochemical Impedance Spectra

**Figure 9:**
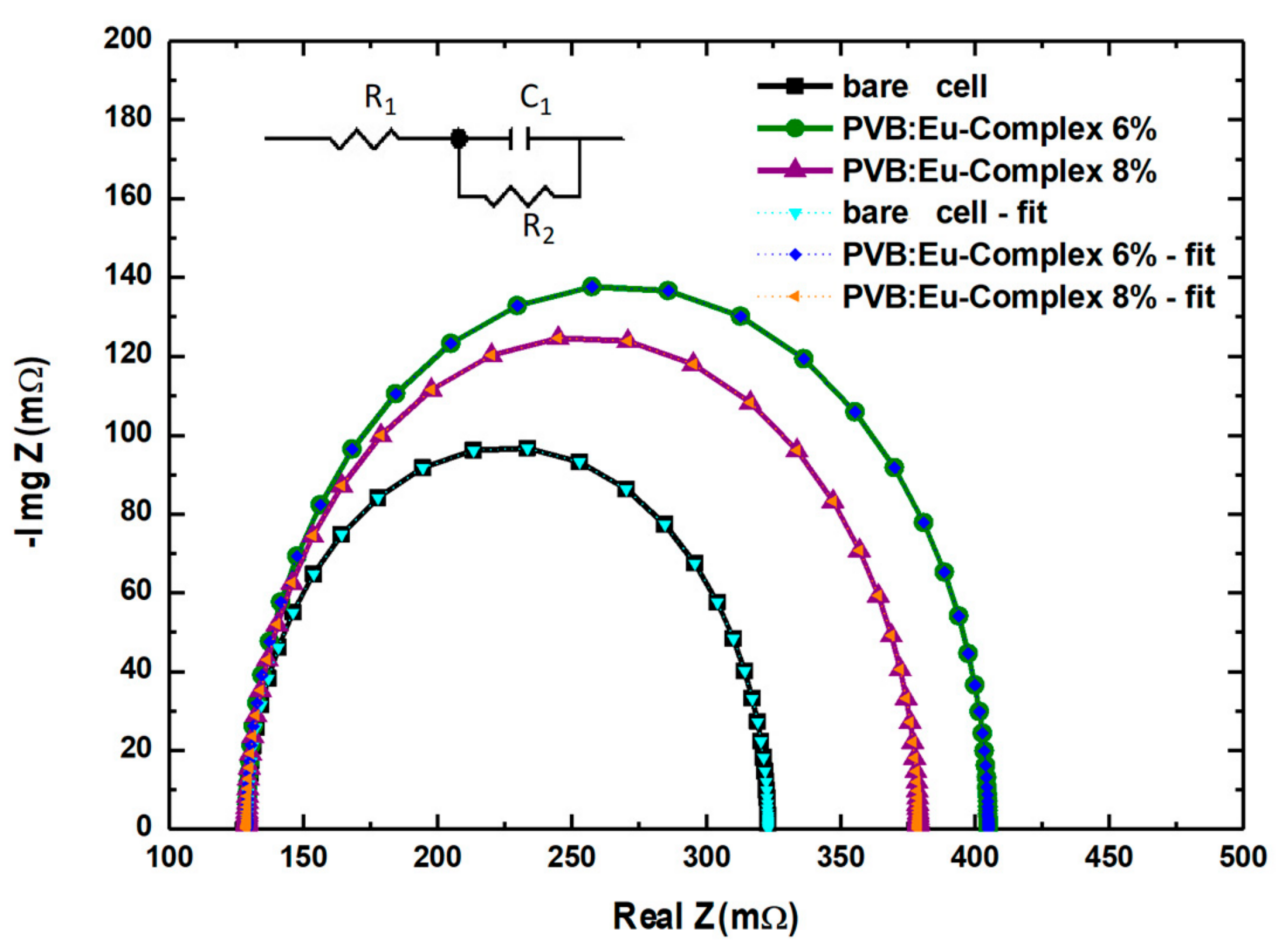
Nyquist plot under illumination conditions (under irradiation of standard AM1.5G) of different concentrations of Eu(^3^DPIQC)_3_ complex and bare c-Si solar cell.

To further understand the charge transport properties of the cells before and after the deposition of the LDSL, Electrochemical Impedance Spectroscopy (EIS) measurements were carried out at their open circuit potential over a frequency range of 10^−3^–10^5^ Hz by means of a OrigaFlex BiPotentiostat. Figure 9 displays a Nyquist plot illustrating the experimental results for the pristine solar cells, as well as for those with LDSLs containing 6% and 8% of the Eu complex. The observed semicircles have been carefully fitted to the equivalent circuit (RC), as depicted in the box, utilizing the ZView 3.2b software. These spectral patterns are consistent with previous literature reports that typically show a single semicircle for this type of solar cell [54,55].

Within this equivalent circuit, RC serves to determine the resistance related to the contributions of the external circuit (R_1_) and the recombination resistance (R_2_) in parallel with the junction capacitance (C_1_).

To understand the results, we have applied the theory of total impedance:

(2) $$Z\left(\omega \right)=Z^{\prime }\left(\omega \right)+jZ\prime \prime (\omega )$$

where *ω* represents the signal frequency for each studied cell and *Z*′ and *Z*″ represent the real and imaginary impedances, respectively. These components take into account the presence of resistive, capacitive, and inductive elements in the real and imaginary parts of the impedance. The real component (*Z*′) and the imaginary component (*Z*″) of the impedance are defined as follows:

(3) $$Z^{\prime }\left(\omega \right)=R_{1}+\frac{C_{1}}{1+{\left(\omega R_{2}C_{1}\right)}^{2}}\;$$

(4) $$Z\prime \prime \left(\omega \right)=\frac{\omega R_{2}^{2}C_{1}}{1+{\left(\omega R_{2}C_{1}\right)}^{2}}$$

The EIS analysis results are listed in Table 4. The EIS spectra of the PV devices show that *R*_1_ values were similar for all PV devices under study, measuring 129 mΩ. Conversely, the recombination resistance value (*R*_2_) for the bare cell stood at 200 mΩ. This value increased to 276 mΩ in the cell containing a 6% Eu complex. This increase signifies a more efficient charge transfer at the interface when the complex is added, resulting in reduced recombination losses at the interface, potentially due to an effective blocking effect brought about by the Eu complex.

However, when the percentage of the Eu complex is further increased to 8% in the solar cell, there is a decrease in the semicircle on the Nyquist diagram, indicating an increase in recombination at the interface. This suggests that the cell is affected by interface defects resulting from poor passivation. Consequently, after reaching the optimal value for films with 6% for the Eu complex, the cell’s photoconversion efficiency decreases.

Moreover, the junction capacitances (C_1_) for the cells also experienced a minor increase when including the LDSL. This trend further reinforces the notion that the optimal performance for the cell occurs with 6% of the Eu complex. These findings align with other experimental results, such as short-circuit currents, open-circuit potential, and the quantum efficiency of the cells. These results collectively indicate that the addition of the Eu complex at 6% is optimal for enhanced light collection and improved solar cell performance.

All measurements were carried out under illumination conditions (AM solar spectrum 1.5G illumination of one sun (100 mW/cm^2^)).

## 3. Materials and Methods

### 3.1. Characterization Techniques

The infrared spectrum of the Eu(^3^DPIQC)_3_ complex was recorded using a Perkin-Elmer FT-IR Spectrometer Spectrum Two (Llantrisant, UK) with KBr pellets. NMR spectra were recorded in CDCl_3_ at 500 or 700 MHz (Bruker Advance III, Oxford, UK). Chemical shifts were reported in parts per million (*δ*) using the residual solvent signals as an internal standard for ^1^H and ^13^C NMR spectra and coupling constants (*J*) in Hz. The Raman spectrum was recorded using the Raman Jasco RNS-4500 spectrometer (Easton, MD, USA). Mass spectra (ESI-MS) were acquired using an Agilent 1200 ESI/APCI QTof tandem Agilent Mass QTof 6520 (Santa Clara, CA, USA). The powder X-ray diffraction (PXRD) data were collected using a Bruker D8 Advance diffractometer (Billerica, MA, USA) fitted with a graphite monochromator using CuK_α_ (λ = 1.54057 Å) radiation in the range 10° ≤ 2θ ≤ 70°, operated at 40 kV and 30 mA. The atomic force microscopy (AFM) images were recorded on a Witec CRC200 microscope. The absorption spectra were measured in a Jasco V-770 spectrophotometer (Easton, PA, USA) equipped with an integrating sphere ISN-923. Excitation and emission spectra were recorded using a Jasco FP-8500 spectrofluorometer (Easton, PA, USA), and all measurements were carried out at room temperature. To ensure the comparability of photoluminescence intensities, the sample weight was kept consistent across all experiments.

### 3.2. Computational Modelling

In order to gain some deeper knowledge of the optical properties of Eu(^3^DPIQC)_3_, we used the lanthanide luminescence software (LUMPAC, v.1.4.1) [35] to compute its most relevant photophysical parameters. The molecular structure has been optimized using the RM1/Sparkle model implemented in MOPAC [36,56], taking the crystal structure provided by Cai et al. [28] as a starting point. The ligand-centered excited states for the Eu(^3^DPIQC)_3_ complex in its optimized geometry were obtained from semiempirical ZINDO/S calculations using the ORCA program (v.4.2.1) [57,58]. In these calculations, the central Eu^3+^ cation is conveniently replaced by a simple +3e point charge.

Judd–Ofelt theory serves as a valuable tool for the analysis of f-f inner shell electronic transitions. Within Judd–Ofelt theory, the dipole strengths are described by three phenomenological parameters: Ω_λ_ (λ = 2, 4, 6) [44,46,59]. The experimental Ω_λexp_ parameters were derived from the luminescence spectra by correlating the emission intensities to the integrated areas under the emission bands in the luminescence spectrum using LUMPAC. Theoretical intensity parameters Ω_λcalc_ have also calculated from the geometry obtained from the optimized RM1/Sparkle model [60]. Energy transfer rates have been obtained using the model proposed by the group of O. L. Malta [61].

The shape and symmetry of the coordination environment of the Eu^3+^ cation has been characterized by means of continuous shape and symmetry measures using the Cosymlib Python library [62,63].

### 3.3. Preparation of the Polymeric Luminescent Films

The Eu(^3^DPIQC)_3_ complex was incorporated into the polymer matrices via the spin-coating method. Initially, the polymer, polyvinyl butyral (PVB), was dissolved in CH_2_Cl_2_ (1.0 g/6 mL). Subsequently, different amounts of europium complex EuL_3_ (2, 4, 6, 8% *w*/*w*) were added to the polymer solution under stirring at rt for a duration of 30 min. Finally, the LDSL films were coated on glass substrates using the spin-coating method [64]. A spin coater model SPS Polos Spin 150i (Putten, The Netherlands) was used for this purpose. For deposition, 2.5 mL of Eu(^3^DPIQC)_3_/PVB/CH_2_Cl_2_ mixture was taken and deposited on the optical glass (K9 glass with an area of 30 mm × 30 mm and thickness of 3 mm) to be studied. The spin-up was performed with a rotation speed of 200 rpm and an accelerated rotation at 1000 rpm/s with a 5 s interval. The spin-off was performed with a rotational speed of 3000 rpm, with accelerated rotation at 10,000 rpm/s in a rotation time of 20 s. The edge bead removal was performed with a rotation speed of 600 rpm and an accelerated rotation at 1000 rpm/s in a 10 s interval. The drying was performed with a rotation speed of 4000 rpm and an accelerated rotation at 1000 rpm/s in a 20 s interval.

### 3.4. Device Fabrication and Characterization

Passivated emitter rear totally diffused (PERT) solar cells were fabricated using n-type phosphorus-based monocrystalline Si <100> wafers (Czochralski) with dimensions of 244.31 cm^2^, a thickness of 180 μm, and a resistivity ranging from 5 to 5.9 Ωcm. The fabrication process involved several steps, as follows:

1. Wet chemical alkaline saw damage etching: The wafers underwent a wet chemical alkaline etching process to remove the damage caused by sawing. Subsequently, they were thoroughly cleaned.
2. P-doping profiles for the n+ type back surface field (BSF): The sample was prepared using POCl_3_ diffusion in a quartz tube furnace. This profile, denoted as P-BSF, achieves a surface concentration of 6 × 10^19^ cm^−3^ with a junction depth of approximately 0.45 μm.
3. Silicon nitride (SiN_x_) deposition: After the POCl_3_ diffusion process for the BSF, a silicon nitride layer was deposited on the rear side using plasma-enhanced chemical vapor deposition (PECVD).
4. Wet chemical alkaline texturing: The wafer was subjected to a wet chemical alkaline texturing process to enhance light absorption. This step was followed by another cleaning process.
5. Formation of the p-n junction: On the front side, the p-n junction was formed through BBr_3_ diffusion, which created the necessary doping for the formation of the junction.
6. Passivation/antireflection coatings: The passivation and antireflection coatings were applied to the front side of the solar cells. These coatings consisted of stacks made of thermal silicon dioxide and silicon nitride (SiO_2_/SiN_x_). The thermal silicon dioxide layer was achieved through thermal oxidation, while the silicon nitride layer was deposited using PECVD.

By following these fabrication steps, the PERT solar cells were successfully processed, ready for further characterization and performance evaluation. The photovoltaic performance of the n-PERT solar cells, both with and without the LDSL, was evaluated at room temperature by measuring current–voltage (I–V) curves and the external quantum efficiency (EQE). I–V curves were obtained using a solar simulator model CT80AAA from Photo Emission Tech. (Ventura, CA, USA), which provided standard AM solar spectrum 1.5G illumination of one sun (100 mW/cm^2^) using a 300 W Xe lamp. The current–voltage measurements were conducted using a Keithley Source-meter model 2400 (Cleveland, OH, USA). The cell tester was calibrated using a reference c-Si solar cell (PET reference cell model #60909). EQE spectra were measured using a PET Quantum Efficiency System (Ventura, CA, USA) equipped with two light sources for monochromatic illumination, consisting of a 300 W Xenon arc lamp and a halogen lamp.

## 4. Conclusions

Luminescent down-shifting layers (LDSLs) containing different amounts of an Eu(III) complex based on 3-(diphenylphosphoryl)-1-isoquinolinecarboxylic acid embedded within a polyvinyl butyral (PVB) matrix were carefully deposited onto a glass surface using the spin-coating method. To gain a deeper understanding of the absorption bands of the Eu complex in a CH_2_Cl_2_ solution, a computational study was conducted. In this study, shape measures for the coordination polyhedron in both the crystal structure and the optimized geometry were calculated, revealing a moderate deviation from the ideal tricapped-trigonal-prismatic geometry. Continuous symmetry measurements demonstrated that the C_3_ axis remained intact in both the crystal and optimized structures, with S (C_3_ ≈ 0). These results indicate that the coordination environment of Eu is far from being centrosymmetric in both crystal and optimized structures, giving rise to intense f-f transitions. The computed UV-vis spectrum showed a peak at 312 nm, which could be attributed to a π → π* transition within one of the three ^3^DPIQC ligands, leading to the lowest excited singlet state (S_1_) at an energy level of 3.98 eV (32,128 cm^−1^) above the ground state and an efficient transfer to the lowest triplet state T_1_, calculated to lie 2.79 eV (22,485 cm^−1^) above the ground state. The T_1_ state was found to be well aligned for an effective energy transfer between the ligands and the ^5^D_1_ and ^5^D_0_ levels of the central Eu^3+^ cation.

On the other hand, the experimental and calculated Judd–Ofelt parameters for the Eu complex in solution show excellent agreement with those obtained for the complex embedded in PVB. The values of the Ω_4_ parameter were slightly higher in the polymer films compared to the pure complex, suggesting a subtle perturbation introduced by the polymer matrix on the chelate effects of the ligands.

Finally, the LDS layers were utilized as energy converters in c-Si solar cells to enhance the utilization of high-energy photons, thereby improving the overall efficiency of the solar cells. The luminescent films based on the Eu(^3^DPIQC)_3_ complex effectively absorb the UV component of the solar spectrum and convert it into emissions within the visible spectral range. The substantial impact of the LDSL coating on enhancing the performance of c-Si solar cells was evident through the analysis of the External Quantum Efficiency (EQE) curves for both the uncoated and PVB film-coated cells, each containing varying concentrations of the Eu complex. The EQE spectra of the photovoltaic devices displayed an improvement in the spectral response within the UV/blue region, specifically between 300 and 450 nm when utilizing LDS layers. This application can help mitigate the overheating loss of the c-Si cells attributed to UV radiation.

The EIS analysis shows a slight increase in the junction capacitances (C_1_) for the cells. This trend further underscores the notion that the cell performs at its best with the 6% Eu complex concentration. These findings are in line with the outcomes of other experimental measurements, such as short-circuit currents, open-circuit potential, and the quantum efficiency of the cells. Taken together, these results conclusively demonstrate that the incorporation of the Eu complex at a 6% concentration is the optimum choice for achieving enhanced light collection and overall improvement in the performance of the solar cell.

## Figures and Tables

**Table 1:** Solid-state photophysical data for the ^5^D_0_ fluorescence data of the Eu(^3^DPIQC)_3_ complex and the hybrid PVB: Eu(^3^DPIQC)_3_ with different amounts of Eu^3+^. Judd–Ofelt parameters Ω_i_ in 10^−20^ cm^2^, transition probabilities in s^−1^.

Sample	Ω_2_	Ω_4_	Ω_6_	A_RAD_	A_NRAD_
Eu(^3^DPIQC)_3_ (calc.)	4.13	0.49	0.03	166.24	288.30
Eu(^3^DPIQC)_3_ (exp.)	4.14	0.48	-	181.51	273.04
PVB: Eu(^3^DPIQC)_3_ (2%)	4.39	2.00	-	211.52	243.05
PVB: Eu(^3^DPIQC)_3_ (4%)	4.49	1.38	-	205.24	249.30
PVB: Eu(^3^DPIQC)_3_ (6%)	4.60	0.96	-	202.32	255.22
PVB: Eu(^3^DPIQC)_3_ (8%)	4.71	0.90	-	204.95	249.60

**Table 2:** Variation in the photovoltaic parameters for c-Si solar cells coated with Eu complex embedded in PVB as a function of the Eu complex concentration. The average external quantum efficiency (EQE) values determined for three different wavelength ranges are also listed. All measurements were carried out at room temperature.

Eu Complex Concentration (%)	*J_sc_* (mA/cm^2^)	V_oc_ (mV)	FF (%)	∆η(%)	${∆\eta }_{LDSL}$ (%)	EQE (%) 300–330 [nm]	EQE (%) 330–400 [nm]	EQE (%) 400–1000 [nm]	J_sc_ cal. (mA/cm^2^)
0	30.44	600.0	69%	12.60	--	0.02	34.27	86.57	35.28
2	30.56	600.7	69%	12.67	0.6	0.67	34.48	86.77	35.43
4	30.50	600.2	69%	12.63	0.2	1.11	34.17	86.43	35.30
6	31.10	600.9	69%	12.89	2.3	2.26	32.23	85.76	40.17
8	30.43	599.9	69%	12.60	0.0	2.69	30.11	85.27	39.89

**Table 3:** EQE values for some Eu^3+^-based luminescent downshifting coatings.

LSC Layer	EQE Increase	Ref.
Eu-doped phosphor/SiO_2_	8%	[50]
[Eu(tta)_3_(tppo)_2_]/EVA	19%	[51]
[EuL_3_]/EVA	15%	[51]
[Eu(tfc)_3_:EABP] 1:1 EVA	5%	[52]
[Eu(tfc)_3_/Eu(dbm)_3_phen]/PVA	5%	[53]
Eu(^3^DPIQC)_3_/PVB	8%	This work

EVA = ethylene-vinyl acetate; PVB = Polyvinyl butyral; L_3_ = triazole-pyridine-bistetrazolate; Eu(tfc)_3_ = tris[3-trifluoromethylhydroxymethilene)-d-camphorate]Eu^3+^; EABP = 4,4′-bis (diethylamino)benzophenone; Dbm = dibenzoylmethane; ^3^DPIQC = 3-(diphenylphosphoryl)-1-isoquinolinecarboxylate.

**Table 4:** The EIS spectroscopy analysis of the PV devices with different contents of Eu^3+^.

Sample	*R*_1_ (mΩ)	*R*_2_ (mΩ)	C1(F) × 10^−8^
Bare Cell	129	200	1.98
PVB: Eu(^3^DPIQC)_3_ (6%)	129	276	2.00
PVB: Eu(^3^DPIQC)_3_ (8%)	129	250	1.99

## Data Availability

Data are contained within the article and Supplementary Materials.

## Supplementary Materials

The following supporting information can be downloaded at: https://www.mdpi.com/article/10.3390/molecules28237924/s1, synthesis procedures and characterization of compounds **2**, **3**, **4**, H^3^DPIQC, and Eu(^3^DPIQC)_3_. Copies of IR, ^1^H, ^13^C, and ^31^P-NMR, Raman, and HRMS-ESI spectra, references [65,66].
