# Supplementary material for: Enhancing c-Si Solar Cell Efficiency in the UV Region: Photophysical Insights into the Use of Eu3+ Complexes for Down-Shifting Layer Applications
Source: Molecules. 2023 Dec 4;28(23):7924. doi: 10.3390/molecules28237924 (PMC10708391; doi:10.3390/molecules28237924)
Supplement: Supplementary file 1 [file molecules-28-07924-s001.zip › molecules-2697349-supplementary.pdf]

Electronic Supplementary Information

Enhancing c-Si Solar Cell Efficiency in the UV Region: Photophysical Insights into the Use of Eu3+ Complexes for Down-Shifting Layer Applications

Fabian Vargas 1, Ronald Nelson 1, Dario Espinoza 1, Ivan Brito 2, Laura Sánchez-Muñoz 3, Pere Alemany 3, Sergio Ortiz 4, Pablo Ferrada 5, Alifhers Mestra 1,6 and Jaime Llanos 1,*

1 Departamento de Química, Universidad Católica del Norte, Avda. Angamos 0610, Antofagasta 1270709, Chi-le; fabian.vargas@alumnos.ucn.cl (F.V.); rnelson@ucn.cl (R.N.); despinoza01@ucn.cl (D.E.); alifhers.mestra@ucn.cl (A.M.)

2 Departamento de Química, Universidad de Antofagasta, Antofagasta 1240000, Chile; ibrito@uantof.cl

3 Departamento de Ciència de Materials i Química Física, Institut de Química Teòrica i Computacional (IQTCUB), Universitat de Barcelona, Diagonal 647, 08028 Barcelona, Spain; sanchezlaura124@gmail.com (L.S.-M.); p.alemany@ub.edu (P.A.)

4 UMR 7200 Laboratoire d'Innovation Thérapeutique, CNRS, Strasbourg Drug Discovery and Development In-stitute (IMS), Université de Strasbourg, 67400 Illkirch-Graffenstaden, France; ortizaguirre@unistra.fr

5 Centro de Desarrollo Energético Antofagasta, Universidad de Antofagasta, Angamos 601, Antofagasta 1240000, Chile; pablo.ferrada.m@uantof.cl

6 Centro Lithium I+D+i, Universidad Católica del Norte, Avenida Angamos 0610, Antofagasta 1270709, Chile

* Correspondence: jllanos@ucn.cl; Tel.: +56-55-2355624

Contents

1. Synthesis.. 2

2. Optimization of C-P cross coupling. Synthesis of isoquinoline 4. 6

3. Powder Diffraction Patters.. 7

4. NMR, IR, Raman and MS Spectra 8

1. Synthesis

1.1 General Procedures

All solvents and reagents were purchased from Sigma Aldrich, Merck, or AK Scientific and were used without further purification. The reactions were monitored by thin-layer chromatography (TLC). TLC was performed on silica gel plates and components were visualized by observation under UV light, and/or by treating the plates with oleum solutions, followed by heating. Flash chromatography was carried out on silica gel (63-200 µm) unless otherwise stated. Melting points were determined using a Stuart SMP3 apparatus and were uncorrected. Infrared spectra were measured using a Perkin-Elmer FT-IR Spectrometer Spectrum Two with KBr pellets. NMR spectra were recorded in $CDCl_3$ at 500 or 700 MHz (Bruker Advance III). Chemical shifts were reported in parts per million (*δ*) using the residual solvent signals as an internal standard for ^1H and ^{13}C NMR spectra and coupling constants (*J*) in Hz. The Raman spectrum was recorded using the Raman Jasco RNS-4500 spectrometer. Mass spectra (ESI-MS) were acquired using an Agilent 1200 ESI/APCI QTof tandem Agilent Mass QTof 6520.

1.1.1 Synthesis of methyl isoquinoline-1-carboxylate (2)

To a solution of isoquinoline-1-carboxylic acid (3.0 g, 17.3 mmol, 1.0 equiv.) in MeOH (173 mL), H_2SO_4 (98%, 9.3 mL, 173.24 mmol, 10.0 equiv.) was added as a single portion. The solution was stirred for 24 h at 65 °C. After substrate consumption, the reaction mixture was cooled and neutralized with $NaHCO_3$ (21 g). The precipitate was filtered out and the solvent was concentrated in vacuo. The residue was dissolved in H_2O and extracted with CH_2Cl_2 (2× 50 mL). The combined organic layers were dried over Na_2SO_4, filtered and concentrated in vacuo to give the methyl isoquinoline-1-carboxylate (**2**) as a pale-yellow oil (2.25 g, 69%). $\mathbf{R_f}$ = 0.52 (50% EtOAc/hexane). **IR** (cm^{-1}) *ν*: 3058, 2953, 1724, 1281, 1256, 1140 (Fig. S3). **^{1}H NMR** (700 MHz, $CDCl_3$) δ 8.79 (d, *J* = 8.7 Hz, 1H, H9), 8.59 (d, *J* = 5.6 Hz, 1H, H3), 7.83 (d, *J* = 8.3 Hz, 1H, H6), 7.78 (d, *J* = 5.7 Hz, 1H, H10), 7.69 (t, *J* = 7.0 Hz, 1H, H2), 7.65 (t, *J* = 7.3 Hz, 1H, H1), 4.06 (s, 3H, H14) (Fig. S2). **^{13}C NMR** (176 MHz, $CDCl_3$) δ 166.29 (CO, C11), 148.17 (C, C7), 141.57 (CH, C9), 136.98 (C, C5), 130.64 (CH, C2), 128.87 (CH, C1), 127.17 (CH, C6), 126.91 (C, C4), 126.41 (CH, C10), 124.39 (CH, C3), 53.04 (CH_3, C14) (Fig. S2). **HRMS-ESI** Calculated for $C_{11}H_9NO_2Na$ $[M+Na]^+$: 210.0526, found 210.052 (Fig. S4).

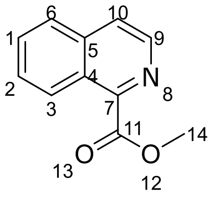

1.1.2 Synthesis of methyl 3-chloroisoquinoline-1-carboxylate (3)

mCPBA (5.2 g, 23.3 mmol, 2.0 equiv.) was added to a solution of methyl isoquinoline-1-carboxylate (2.2 g, 11.6 mmol, 1.0 equiv.) in CH_2Cl_2 (35 mL). The solution was stirred for 18 h at rt. After substrate consumption, the mixture was filtered and washed with $NaHCO_3$ (sat.). The aqueous layer was extracted with CH_2Cl_2 (3× 20 mL). The combined organic layers were washed with H_2O, dried over Na_2SO_4, filtered, and concentrated in vacuo to give the 1-(methoxycarbonyl)isoquinoline 2-oxide as an orange oil (cuant.) which was used without purification in the next step. **R_f** = 0.25 (50% EtOAc/hexane). **IR** (cm^{-1}) *ν*: 3068, 2958, 1744, 1246 (Fig. S5). A solution of 1-(methoxycarbonyl)isoquinoline 2-oxide (2.8 g, 13.9 mmol) in $POCl_3$ (28 mL) was warmed to 105 °C. After 5 h the reaction was cooled at rt, and carefully neutralized with saturated sodium bicarbonate solution. Then, the mixture was extracted with CH_2Cl_2 (3 x 30 mL) and the combined organic layers were washed with H_2O, dried over Na_2SO_4, filtered, and concentrated in vacuo. Purification of the crude material via flash chromatography (SiO_2, 20 to 50% EtOAc/hexanes with 5% CH_2Cl_2) to give chloroisoquinoline **3** as a light brown solid (2.47 g, 80%). **m.p.:** 96-99 °C. **R_f** = 0.60 (50% EtOAc/hexane). **IR** (cm^{-1}) *ν*: 3077, 2955, 1718, 1241, 1150 (Fig. S7). **^{1}H NMR** (700 MHz, $CDCl_3$) δ 8.76 (d, *J* = 8.7 Hz, 1H, H3), 7.90 (s, 1H, H10), 7.82 (d, *J* = 8.3 Hz, 1H, H6), 7.76 (t, *J* = 7.3 Hz, 1H, H2), 7.68 (t, *J* = 7.4 Hz, 1H, H1), 4.09 (s, 3H, H14) (Fig. S6). **^{13}C NMR** (176 MHz, $CDCl_3$) δ 165.38 (CO, C11), 148.78 (C, C9), 144.25 (C, C7), 139.29 (C, C5), 131.74 (CH, C), 129.17 (CH, C1), 126.77 (CH, C6), 126.50 (CH, C3), 125.76 (C, C4), 123.85 (CH, C10), 53.41 (CH_3, C14) (Fig. S6). **HRMS-ESI** Calculated for $C_{11}H_8NO_2ClNa$ $[M+Na]^+$: 244.0136, found 244.0132 (Fig. S8).

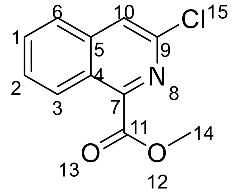

1.1.3 Synthesis of methyl 3-(diphenylphosphoryl)isoquinoline-1-carboxylate (4)

Optimized procedure:[1] A solution of chloroisoquinoline **3** (0.9 g, 4.0 mmol, 1.0 equiv.), diphenylphosphine oxide (1.6 g, 7.9 mmol, 2.0 equiv.), K_3PO_4 (1.7 g, 7.9 mmol, 2.0 equiv.) and $Ni(dppp)Cl_2$ (0.2 g, 0.4 mmol, 10 mol%) in dry xylene (66 mL) under argon atmosphere was warmed to 150 °C. The mixture was stirred for 5 h and after completion of the reaction (the progress of the

[1] See optimization in Table S1 (section 2)

process was monitored by TLC) allowed to cool down to rt and filtered through a pad of Celita® eluting with AcOEt/CH_2Cl_2 (1:1). The filtrate was concentrated in vacuo and purified by flash chromatography (SiO_2, 20% AcOEt/CH_2Cl_2 to 100% CH_2Cl_2) to afford 1.29 g of methyl 3-(diphenylphosphoryl)isoquinoline-1-carboxylate (**4**) as a yellow oil (84% yield). **R_f** = 0.42 (1:2 AcOEt/CH_2Cl_2). **IR** (cm^{-1}) *ν*: 3053, 2953, 1724, 1437, 1236, 1190, 1120 (Fig. S12). **^{1}H NMR** (500 MHz, $CDCl_3$) δ 8.89 (d, *J* = 6.9 Hz, 1H, H3), 8.66 (d, *J* = 7.9 Hz, 1H, H10), 8.02 (m, 5H, H6 + 4xH17), 7.79 (m, 2H, 2xH19), 7.46 (m, 6H, H1 + H2 + 4xH18), 4.06 (s, 3H, H14) (Fig. S9). **^{31}P NMR** (202 MHz, $CDCl_3$) δ 19.15 (s) (Fig. S9). **^{13}C NMR** (126 MHz, $CDCl_3$) δ 166.34 (CO, C11), 149.52 (C, d, *J* = 18.1 Hz, C7), 147.35 (C, d, *J* = 133.8 Hz, C9), 136.16 (C, d, *J* = 10.0 Hz, C5), 132.47 (C, d, *J* = 104.9 Hz, C16), 132.29 (CH, d, *J* = 9.5 Hz, C10), 131.99 (CH, d, *J* = 2.8 Hz, 2xC19), 131.52 (CH, C2), 130.71 (CH, C1), 130.14 (CH, d, *J* = 18.5 Hz, 4xC17), 128.45 (CH, d, *J* = 12.2 Hz, 4xC18), 128.33 (CH, C3), 126.72 (C, d, *J* = 2.6 Hz, C4), 126.43 (CH, C6), 53.07 (CH_3, C14) (Fig. S10). **HRMS-ESI** Calculated for $C_{23}H_{18}NO_3PNa$ $[M+Na]^+$: 410.0917, found 410.0906 (Fig. S13).

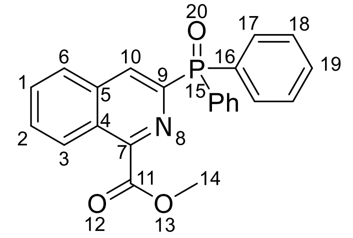

1.1.4 Synthesis of 3-(diphenylphosphoryl)isoquinoline-1-carboxylic acid (H^3DPIQC)

A solution of methyl 3-(diphenylphosphoryl)isoquinoline-1-carboxylate (0.3 g, 0.8 mmol, 1.0 equiv.) and NaOH (60 mg, 1.6 mmol, 2.0 equiv.) in MeOH (2.0 mL)/water (1.0 mL) was stirred for 3 h at 65 °C. When the reaction was complete, the MeOH was concentrated in vacuo, redissolved in $CHCl_3$/H_2O (10 mL, 1:1) and the aqueous layer was separated and acidified with HCl 2 M to pH 2-3. Then, extracted with $CHCl_3$ (3 x 10 mL), dried over Na_2SO_4, filtered, and concentrated in vacuo to give the H^3DPIQC ligand as a white solid (0.26 g, 92%). **m.p.:** 200-202 °C. **R_f** = 0.03 (1:2 AcOEt/CH_2Cl_2). **IR** (cm^{-1}) *ν*: 3048–2225, 1698, 1558, 1439, 1177 (Fig. S16). **^{1}H NMR** (700 MHz, $CDCl_3$[2]) δ 9.53 (m, 1H, H3), 8.89 (d, *J* = 6.8 Hz, 1H, H10), 8.05 (dd, *J* = 6.3, 3.3 Hz, 1H, H6), 7.90 (dd, *J* = 6.4, 3.3 Hz, 2H, 2xH19), 7.80 (dd, *J* = 12.0, 7.9 Hz, 4H, 4xH17), 7.59 (t, *J* = 7.7 Hz, 2H, H1 + H2), 7.50 (td, *J* = 7.7, 2.9 Hz, 4H, 4xH18). **^{13}C NMR** (176 MHz, $CDCl_3$) δ 163.76 (CO, C11), 145.36 (C, d, *J* = 129.8 Hz, C9), 144.87 (C, d, *J* = 15.5 Hz, C7), 137.17 (C, d, *J* = 9.3 Hz, C5), 132.93 (CH, d, *J* = 18.3 Hz, 4xC17), 132.74 (CH, d, *J* = 2.6 Hz, 2xC19), 132.58 (CH, C2), 132.19 (CH, d, *J* = 10.1 Hz, C10), 132,16 (CH,

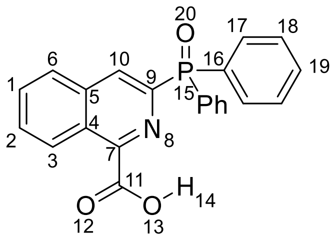

[2] The H14 signal was not observed in $CDCl_3$

C1), 131.07 (C, d, *J* = 107.4 Hz, C16), 128.93 (CH, d, *J* = 12.2 Hz, 4xC18), 128.47 (CH, C3), 127.73 (CH, C6), 127.40 (C, d, *J* = 2.0 Hz, C4). **^{31}P NMR** (162 MHz, $CDCl_3$) δ 23.76 (s) (Fig. S14 y S15). **HRMS-ESI** Calculated for $C_{22}H_{16}NO_3PNa$ $[M+Na]^+$: 396.0760, found 396.0756 (Fig. S17).

1.1.5 Synthesis of the europium (III) complex

A solution of $Eu(NO_3)_3{\cdot}5H_2O$ (38.2 mg, 0.09 mmol, 1.0 equiv.) in MeOH (1.0 mL) was added to a solution of **H^3DPIQC** (0.1 g, 0.3 mmol, 3.0 equiv.) and NaOH (10.7 mg, 0.3 mmol, 3.0 equiv.) in MeOH (1.0 mL) at rt, and a white solid product precipitated immediately. The mixture was filtered and washed with MeOH to afford the HL ligand as a white solid (0.1 g, 88%). **m.p.:** >300 °C. **R_f** = 0.03 (1:2 AcOEt/CH_2Cl_2). **IR** (cm^{-1}) ν: 3056, 1627, 1549, 1358, 1297, 1150, 747 (Fig. S18). **Raman** (cm^{-1}) ν: 3062, 1585, 1352, 999, 409 (Fig. S18). **HRMS-ESI** Calculated for $C_{66}H_{45}N_3O_9EuP_3Na$ $[M+Na]^+$: 1290.1459, found 1290.1423 (Fig. S19).

2. Optimization of C-P cross coupling. Synthesis of isoquinoline 4.

Table S1. Isoquinoline **4** synthesis optimization.[a]

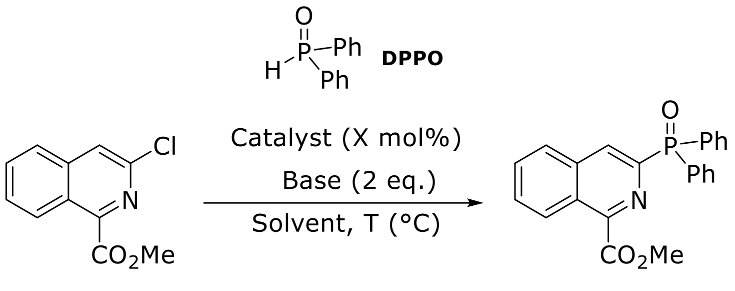

Entry	Catalyst (mol%)	DPPO (eq.)	Solvent	Base	Temp (°C)	Atmosphere	Comp 4 (%)[b]	Reference
1	Ni(dppp)Cl_2 (10)	2.00	Toluene	K_2CO_3	110	N_2	ND	Original conditions[3]
2	Pd(OAc)$_2$ / dppf (5)	1.20	DMF	K_2CO_3	110	N_2	23	[4]
3	Ni(dppp)Cl_2 (5)	1.50	Dioxane	K_2CO_3	100	N_2	4	[5]
4	Ni(dppp)Cl_2 (5)	1.05	DMF	K_2CO_3	50 to 100	N_2	ND	
5	Ni(dppp)Cl_2 (5) / Zn (1 eq)	1.05	DMF	K_2CO_3	100	N_2	ND	
6	Ni(dppp)Cl_2 (10)	2.00	Toluene	K_2CO_3	110	Ar	53	
7	Ni(dppp)Cl_2 (10)	2.00	Xylene	K_2CO_3	150	Ar	74	
**8**	**Ni(dppp)Cl_2 (10)**	**2.00**	**Xylene**	**K_3PO_4**	**150**	**Ar**	**84**	**This work**

[a] *Conditions*: isoquinoine **3** (1.0 eq.) and DPPO (X eq.), were treated with catalyst (X mol%) and Base (2.0 eq.) in the indicated solvent at the indicated temperature (°C), under the indicated inert atmosphere for 12 h. [b] Isolated yield. [c] Diphenylphsphine was detected. ND = Not detecte

[3] Cai, Z., Wei, C., Sun, B., Wei, H., Liu, Z., Bian, Z., Huang, C., 2021. Luminescent europium(iii) complexes based on tridentate isoquinoline ligands with extremely high quantum yield. Inorg. Chem. Front. 8, 41–47. https://doi.org/10.1039/d0qi00894j

[4] Zakirova, G.G., Mladentsev, D.Y., Borisova, N.E., 2019. Palladium-Catalyzed C-P Cross-Coupling between (Het)aryl Halides and Secondary Phosphine Oxides. Synth. 51, 2379–2386. https://doi.org/10.1055/s-0037-1610698

[5] Zhao, Y.L., Wu, G.J., Li, Y., Gao, L.X., Han, F.S., 2012. [NiCl 2(dppp)]-catalyzed cross-coupling of aryl halides with dialkyl phosphite, diphenylphosphine oxide, and diphenylphosphine. Chem. - A Eur. J. 18, 9622–9627. https://doi.org/10.1002/chem.201103723

3. Powder Diffraction Patters

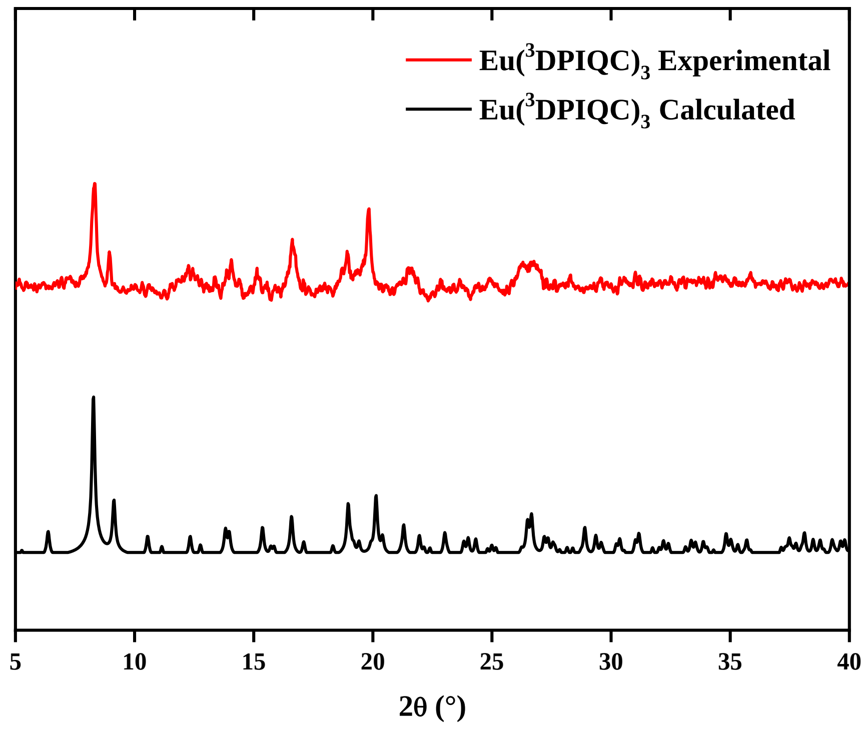

Figure S1. Calculated from the crystallographic data reported by Cai et al.[3]

4. NMR, IR, Raman and MS Spectra

Methyl isoquinoline-1-carboxylate (2)

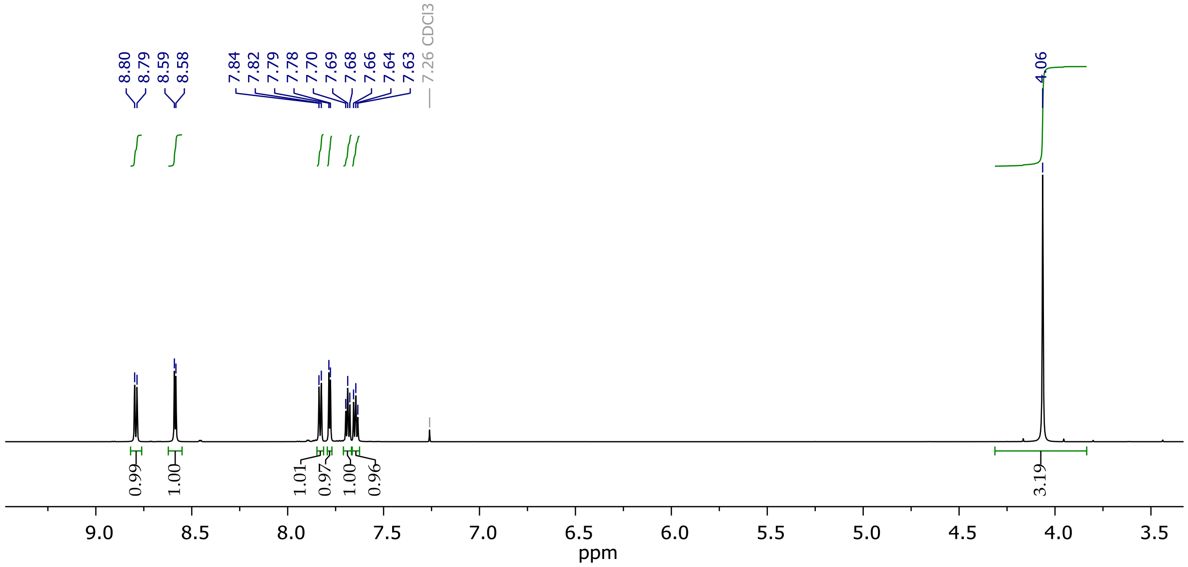

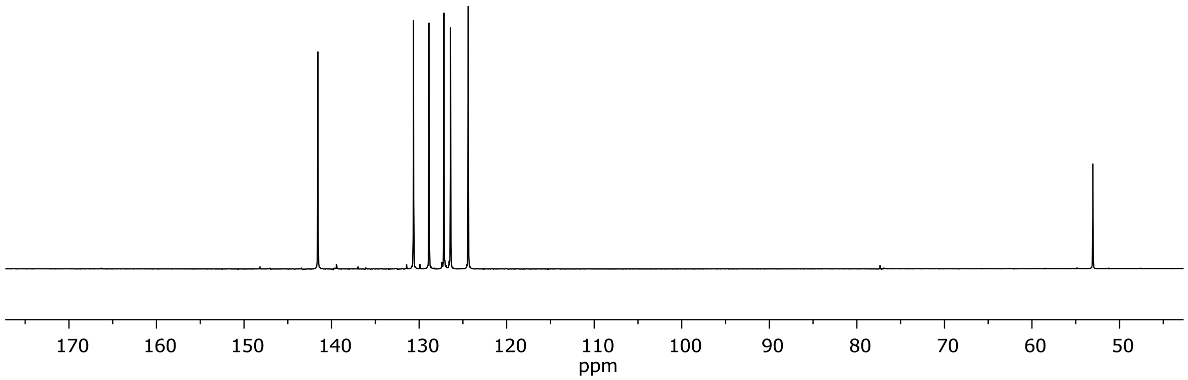

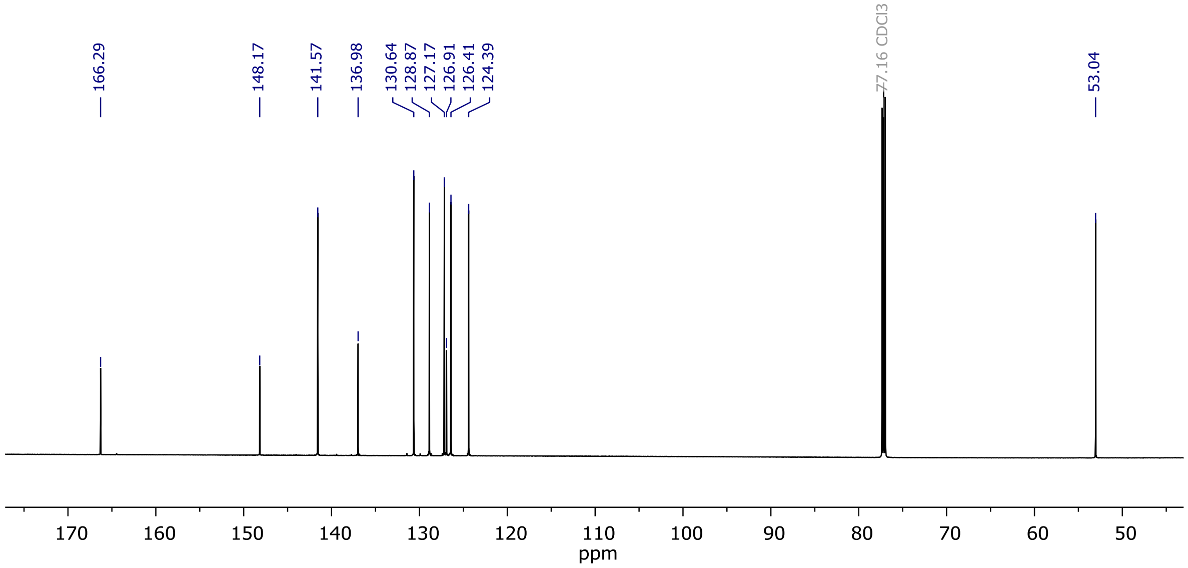

Figure S2 .^{1}H, DEPT-135 and ^{13}C RMN of methyl isoquinoline-1-carboxylate.

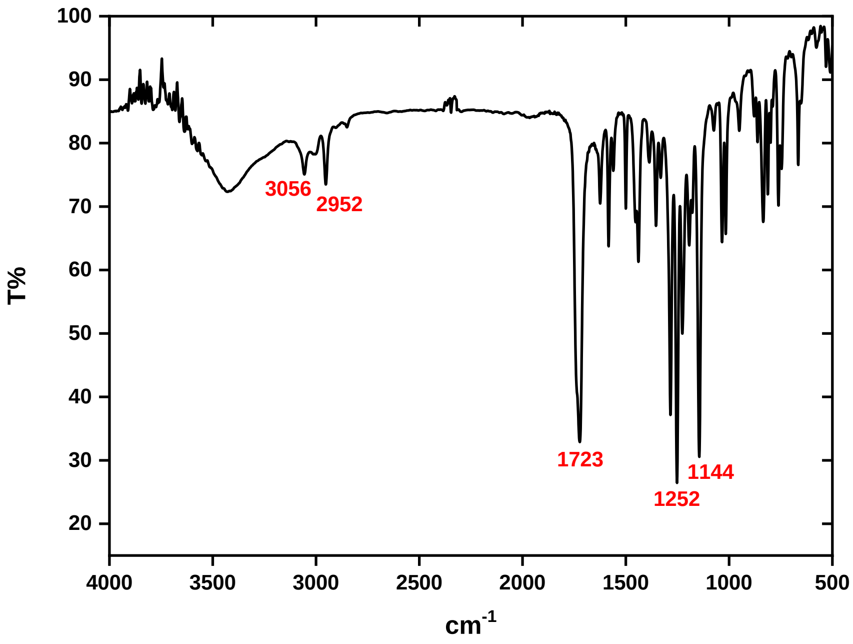

Figure S3. FT-IR spectrum of methyl isoquinoline-1-carboxylate.

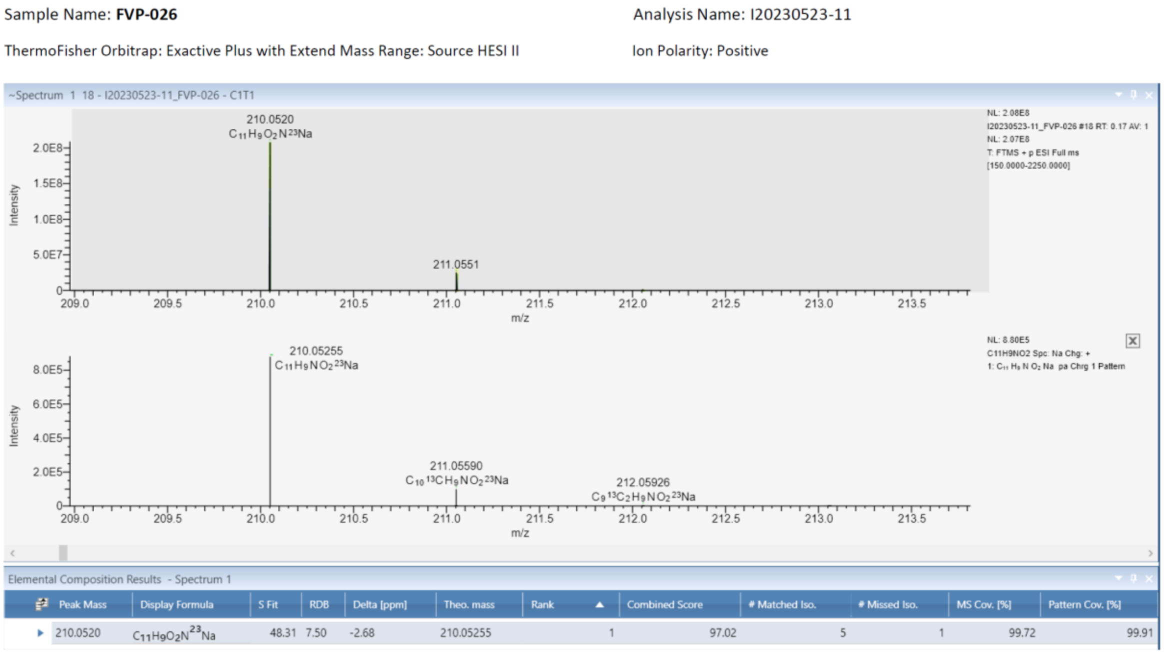

Figure S4 .Found and calculated HRMS-ESI of methyl isoquinoline-1-carboxylate.

1-(methoxycarbonyl)isoquinoline 2-oxide

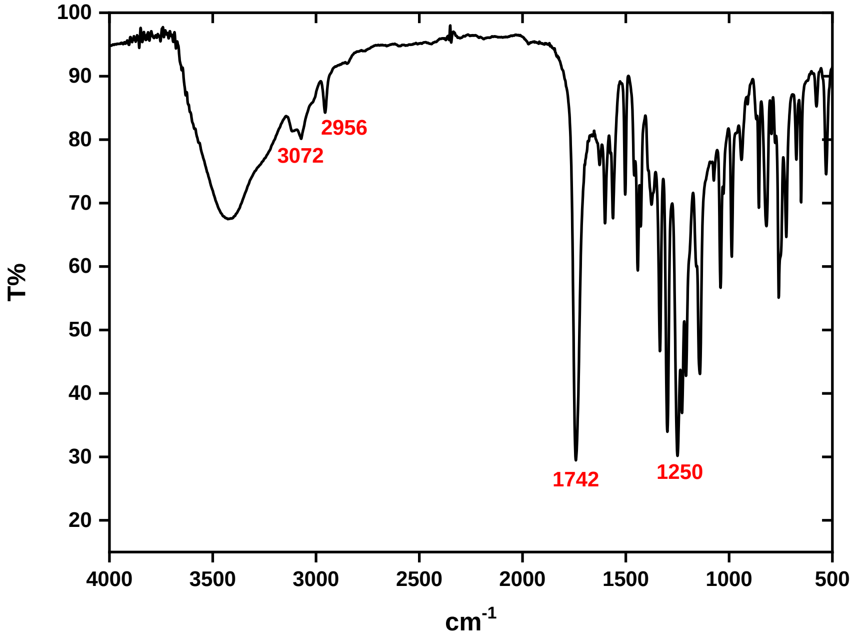

Figure S5 FT-IR spectrum of 1-(methoxycarbonyl)isoquinoline 2-oxide.

Methyl 3-chloroisoquinoline-1-carboxylate (3)

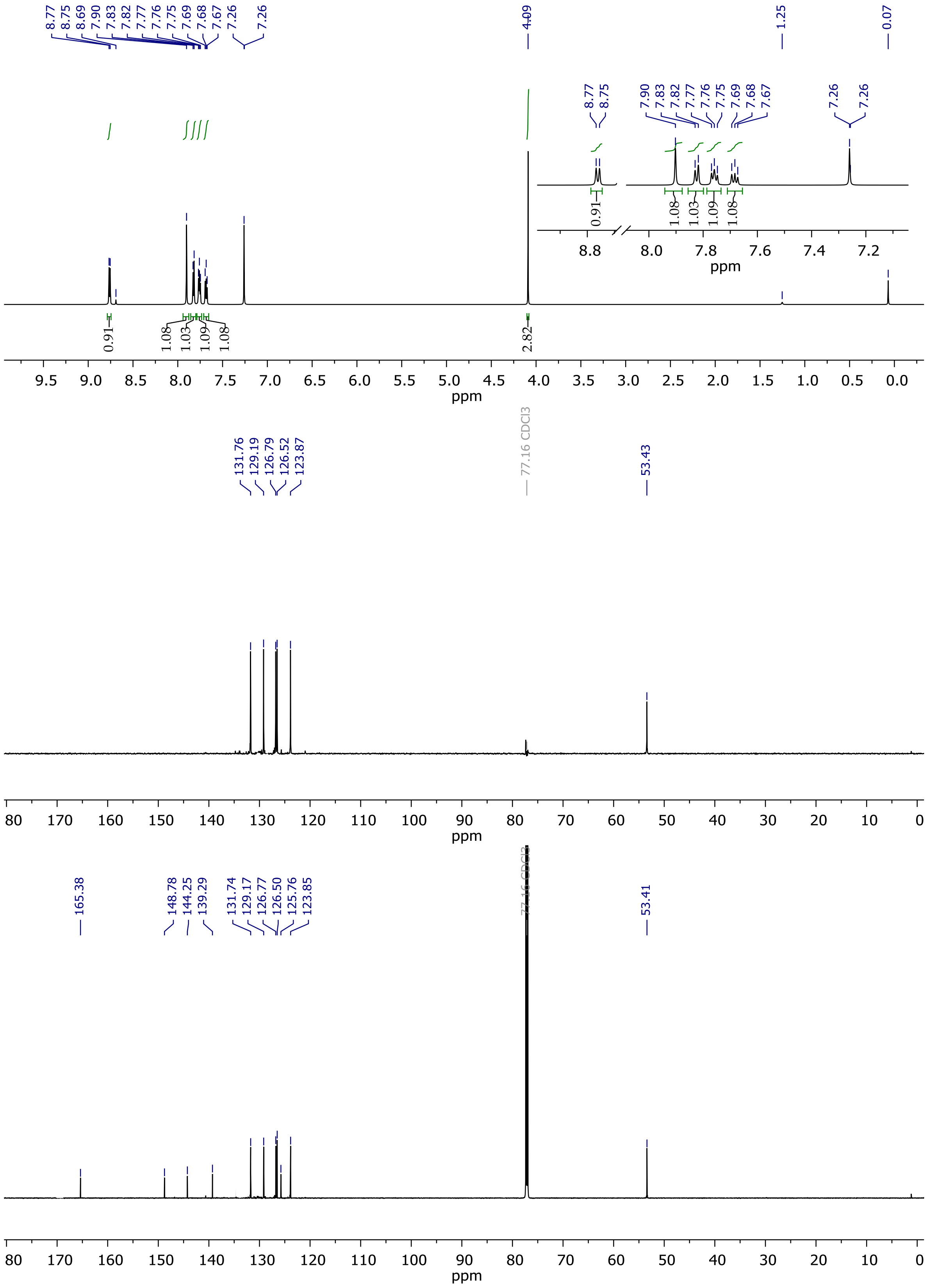

Figure S6 ^{1}H, DEPT-135 and ^{13}C RMN of methyl 3-chloroisoquinoline-1-carboxylate.

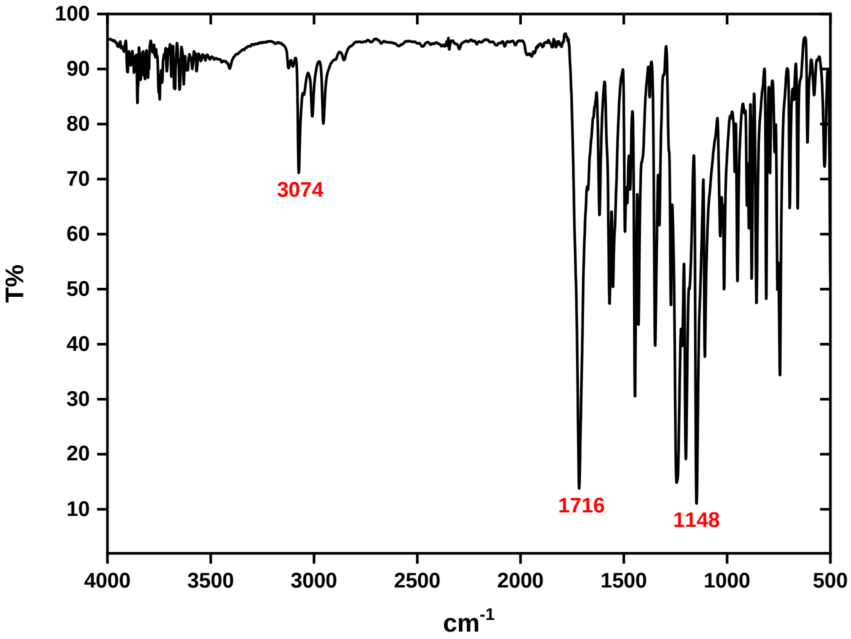

Figure S7 FT-IR spectrum of methyl 3-chloroisoquinoline-1-carboxylate.

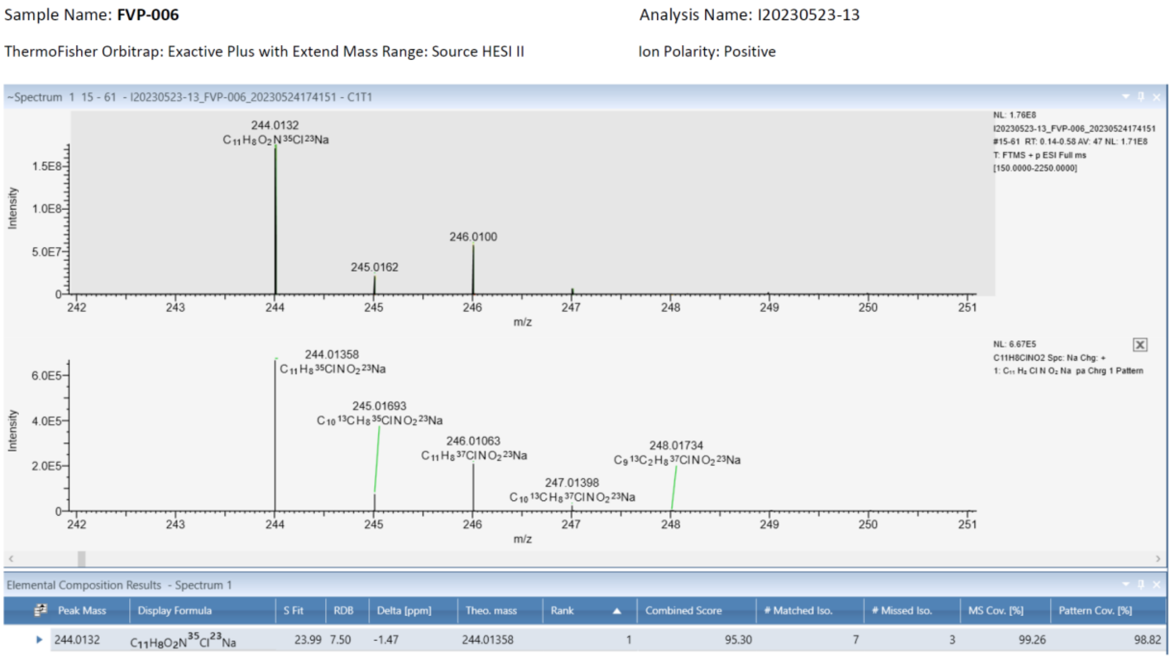

Figure S8 Found and calculated HRMS-ESI of methyl 3-chloroisoquinoline-1-carboxylate.

Methyl 3-(diphenylphosphoryl)isoquinoline-1-carboxylate (4)

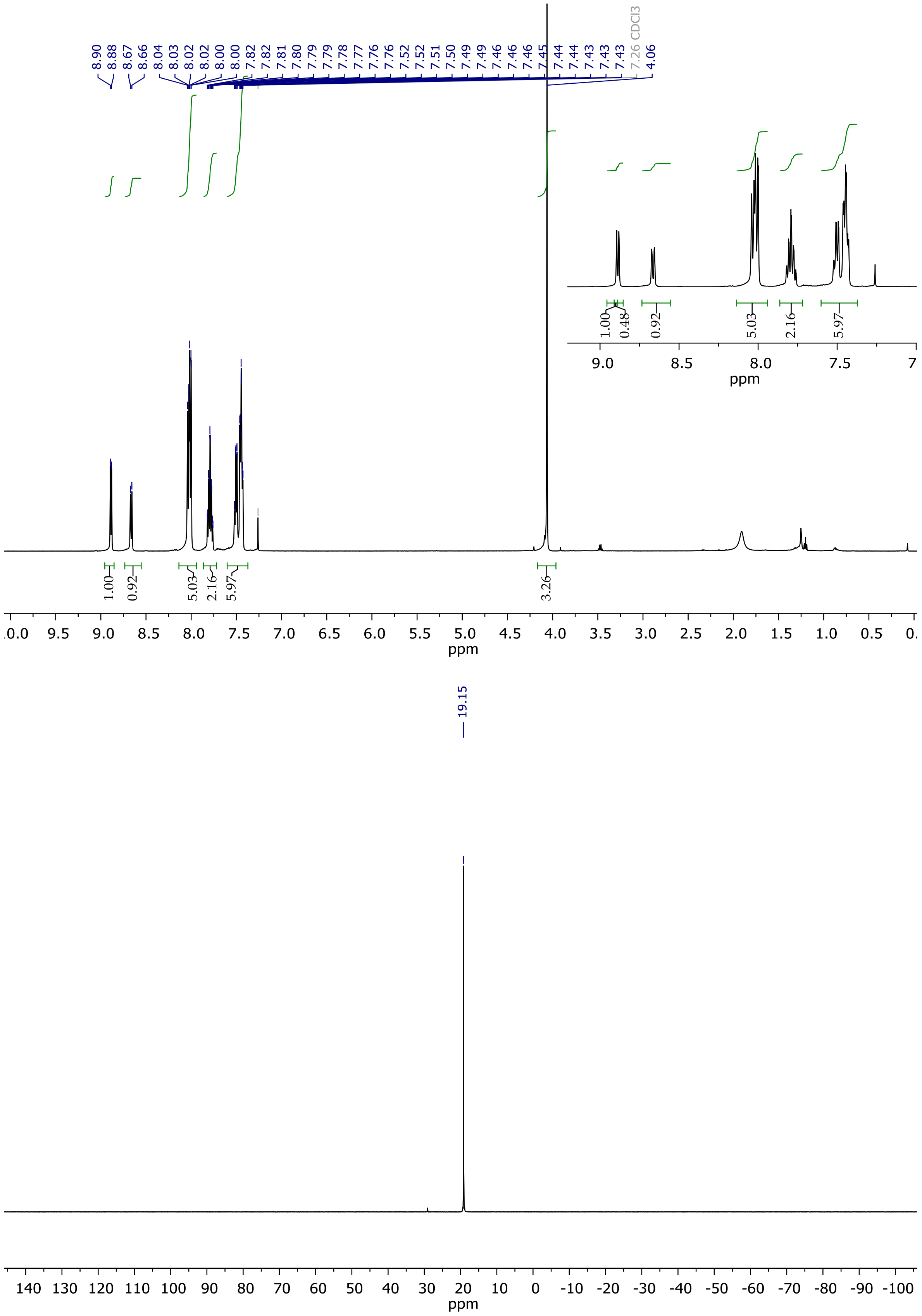

Figure S9 ^{1}H and ^{31}P RMN of methyl 3-(diphenylphosphoryl)isoquinoline-1-carboxylate.

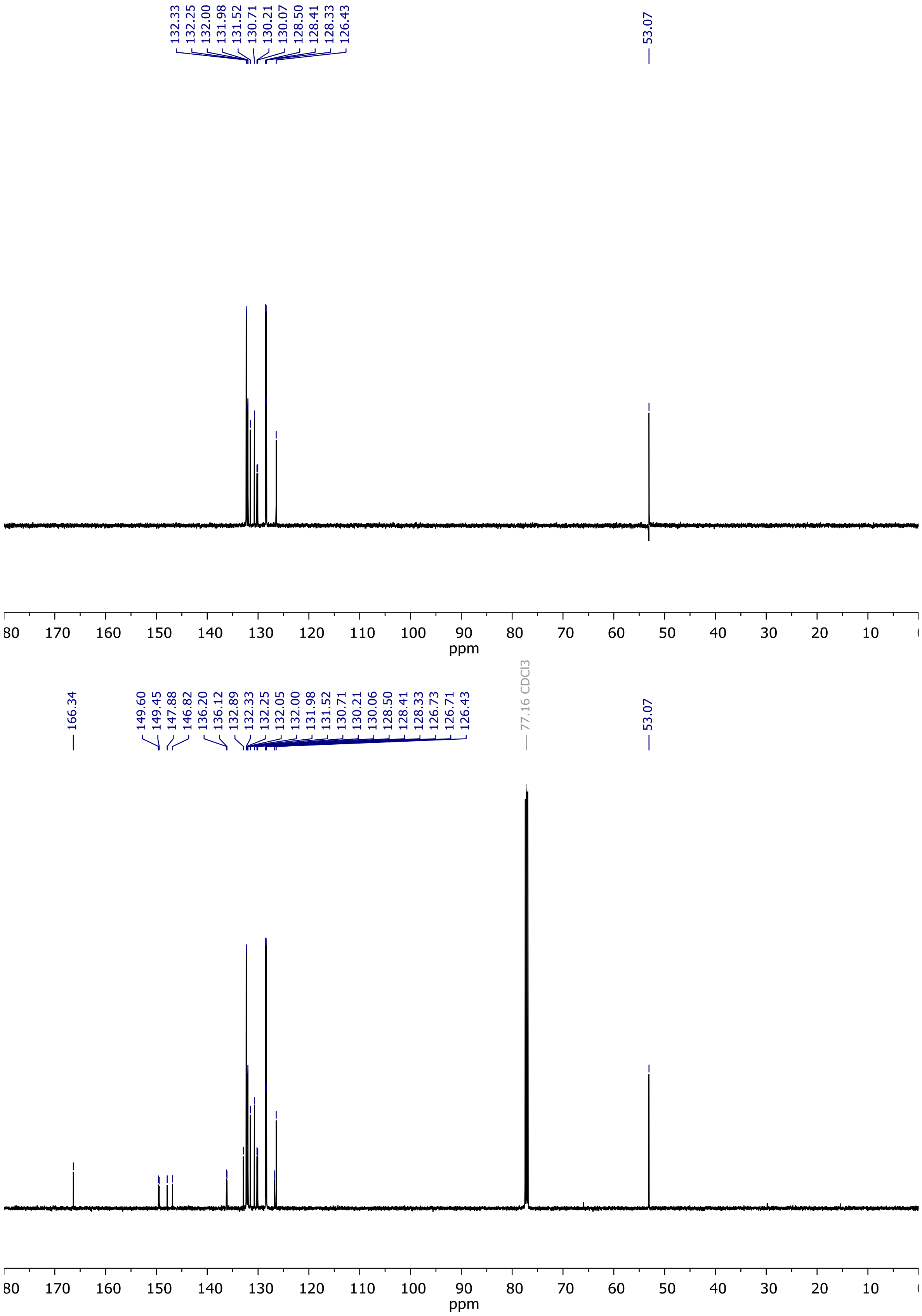

Figure S10 DEPT-135 and ^{13}C RMN of methyl 3-(diphenylphosphoryl)isoquinoline-1-carboxylate.

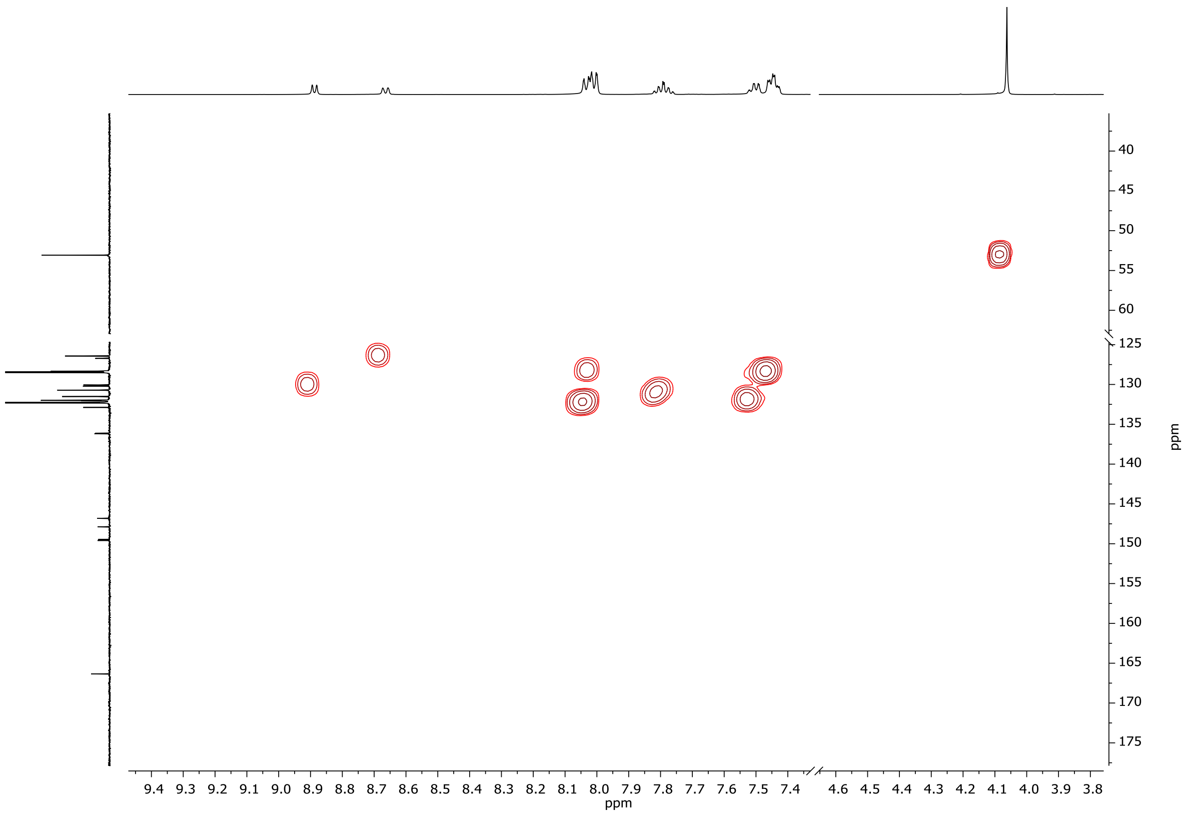

Figure S11 HSQC of methyl 3-(diphenylphosphoryl)isoquinoline-1-carboxylate.

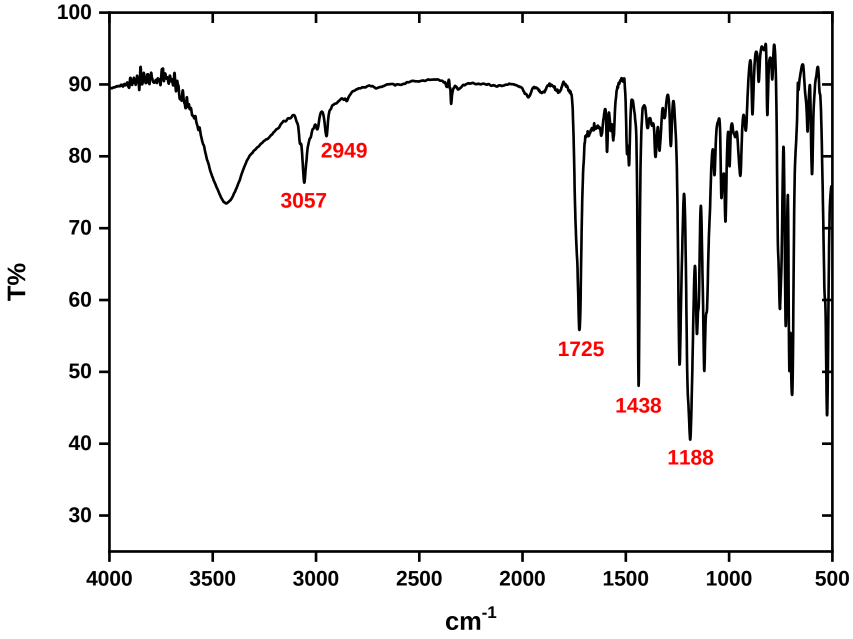

Figure S12 FT-IR spectrum of methyl 3-(diphenylphosphoryl)isoquinoline-1-carboxylate.

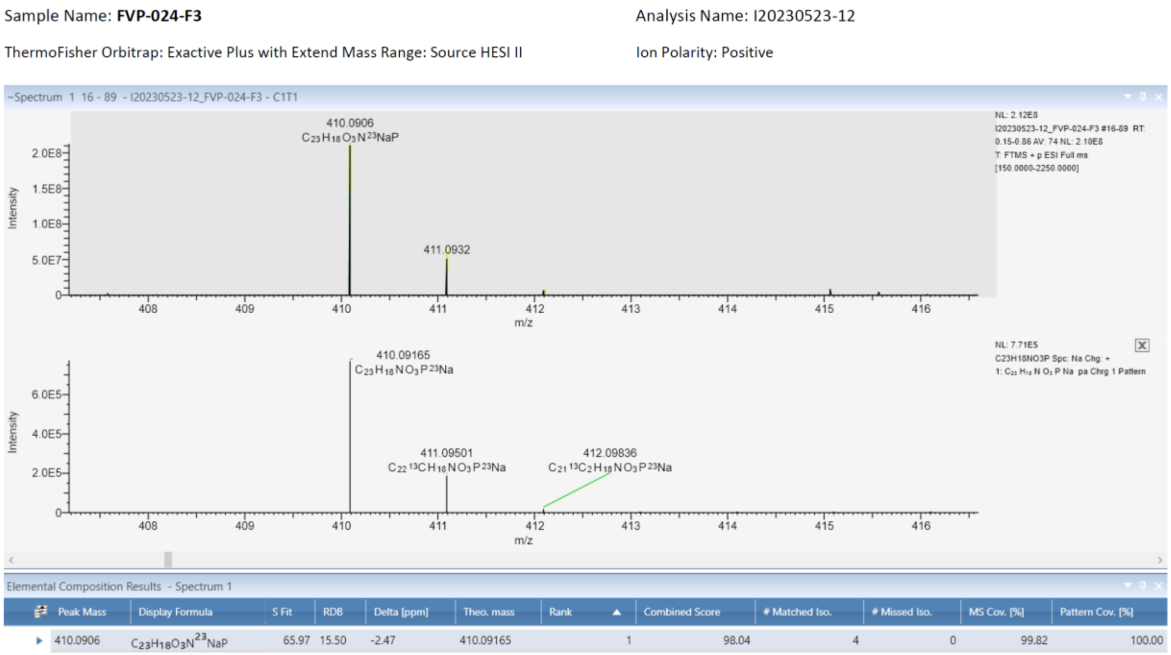

Figure S13 Found and calculated HRMS-ESI of methyl 3-(diphenylphosphoryl)isoquinoline-1-carboxylate.

3-(diphenylphosphoryl)isoquinoline-1-carboxylic acid (H^3DPIQC)

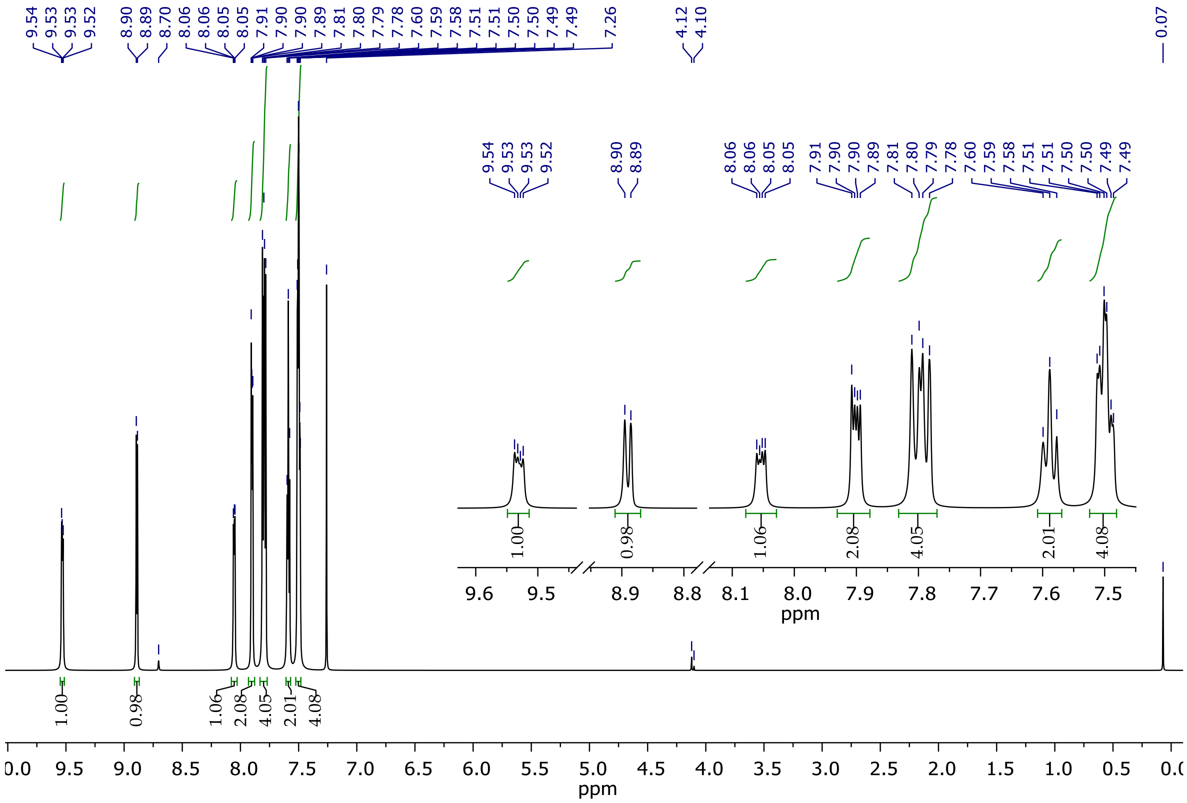

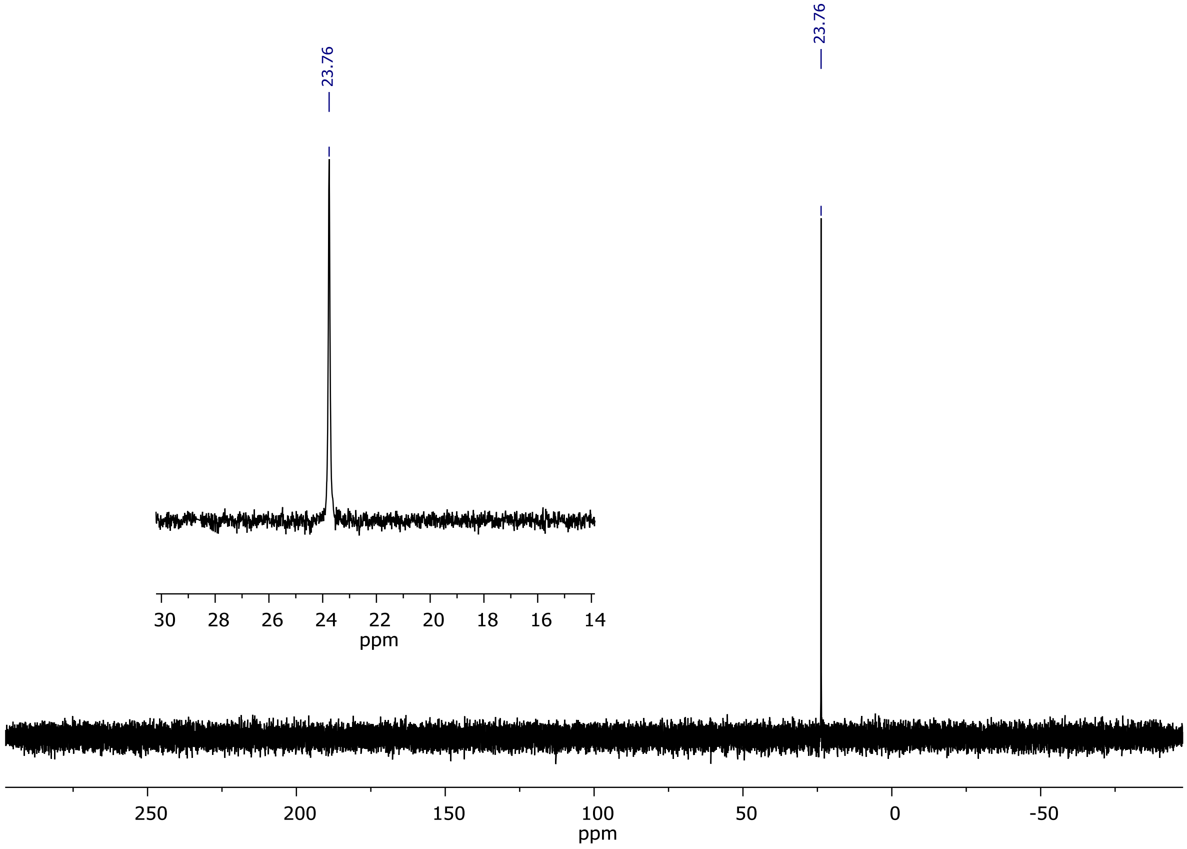

Figure S14 ^{1}H and ^{31}P RMN of 3-(diphenylphosphoryl)isoquinoline-1-carboxylic acid.

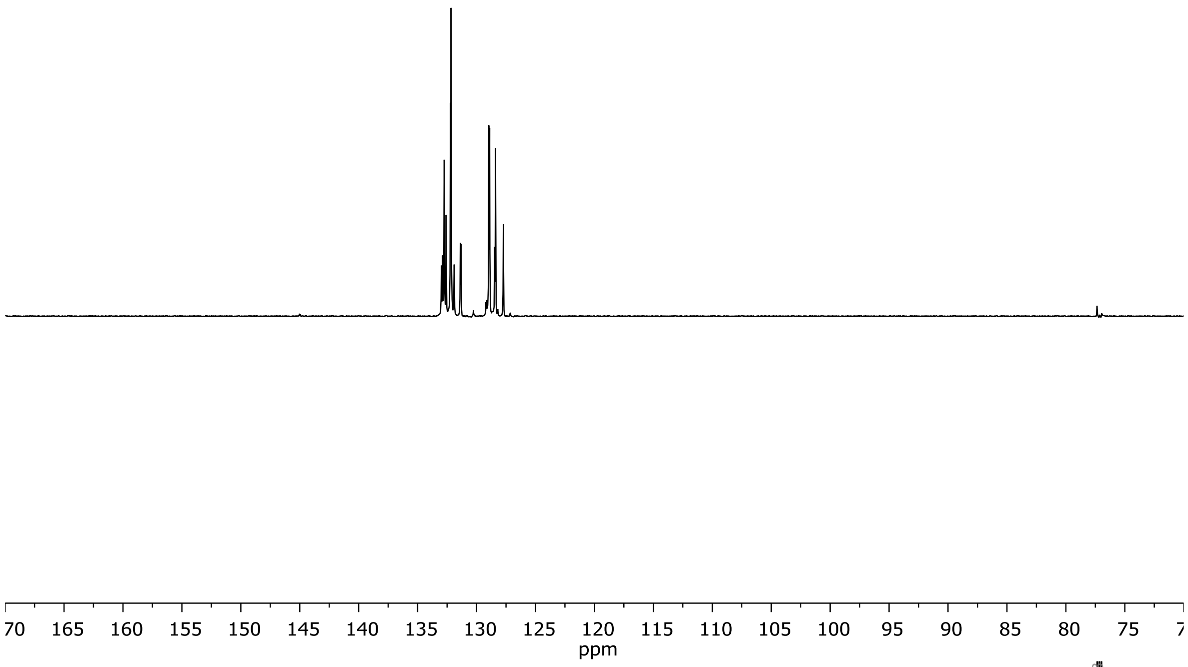

70 165 160 155 150 145 140 135 130 125 120 115 110 105 100 95 90 85 80 75
ppm

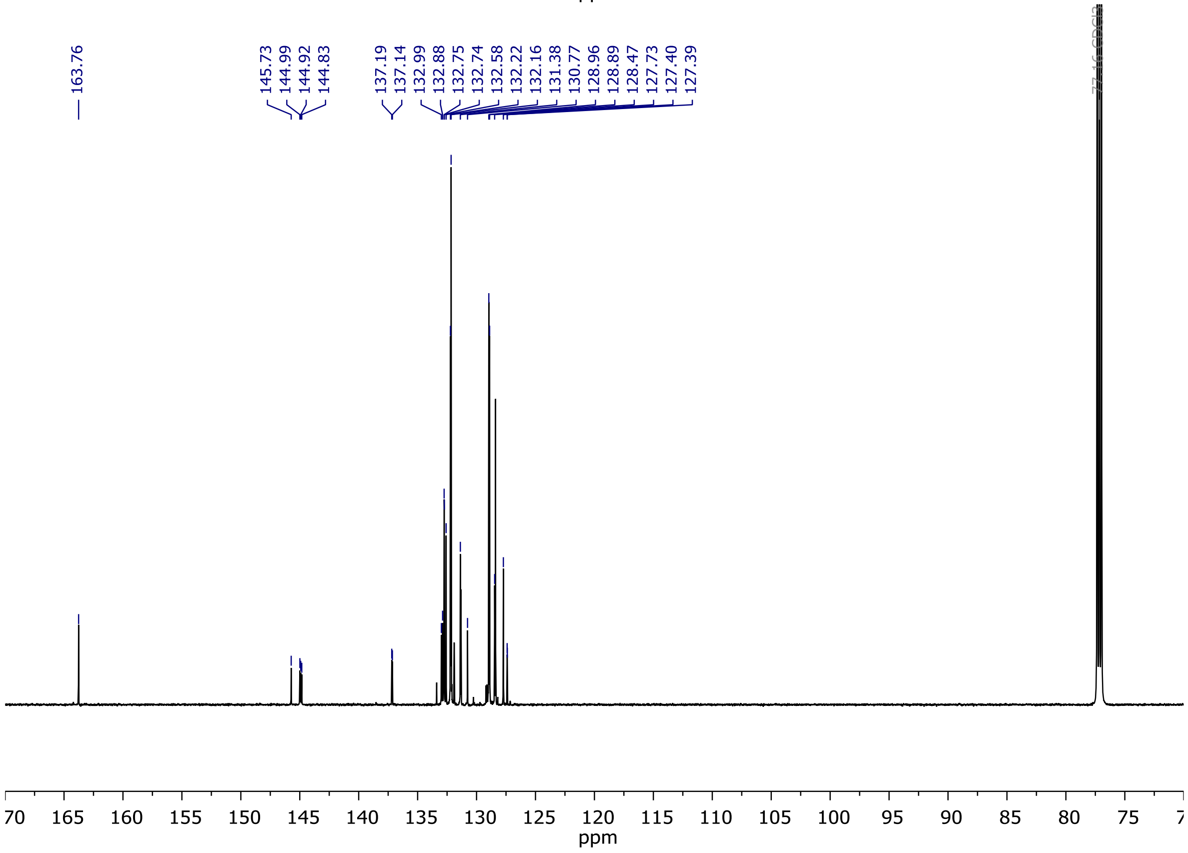

163.76
145.73
144.99
144.92
144.83
137.19
137.14
132.99
132.88
132.75
132.74
132.58
132.22
132.16
131.38
130.77
128.96
128.89
128.47
127.73
127.40
127.39
70 165 160 155 150 145 140 135 130 125 120 115 110 105 100 95 90 85 80 75
ppm

Figure S15 DEPT-135 and ^{13}C RMN of 3-(diphenylphosphoryl)isoquinoline-1-carboxylic acid.

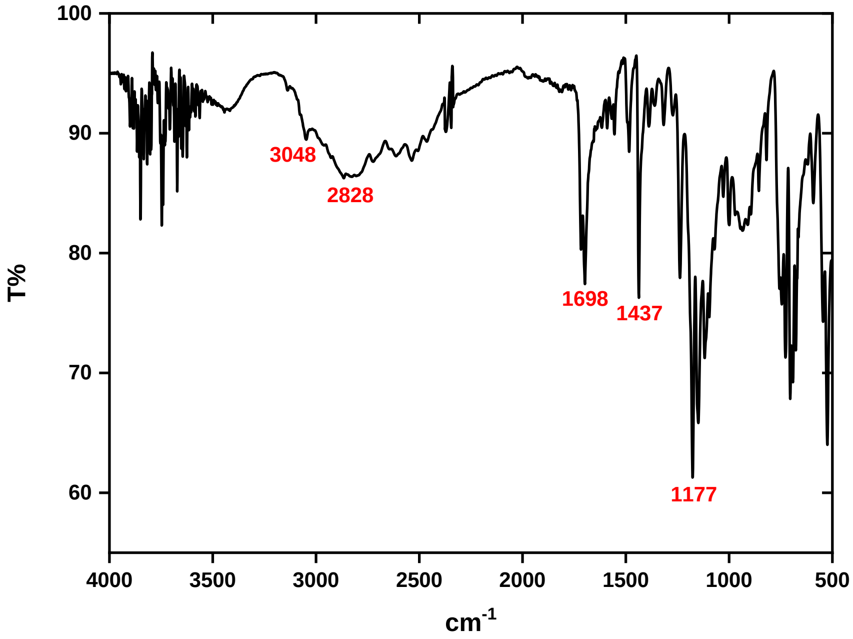

Figure S16 FT-IR spectrum of 3-(diphenylphosphoryl)isoquinoline-1-carboxylic acid.

Sample Name: **FVP-020-F2**

Analysis Name: I20230523-14

ThermoFisher Orbitrap: Exactive Plus with Extend Mass Range: Source HESI II

Ion Polarity: Positive

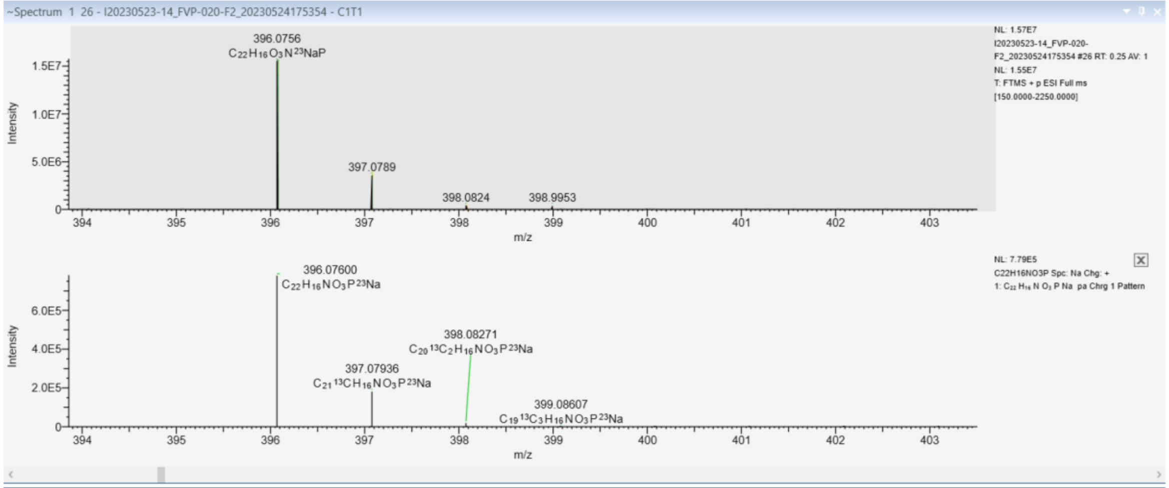

Elemental Composition Results - Spectrum 1

Peak Mass	Display Formula	S Fit	RDB	Delta [ppm]	Theo. mass	Rank	Combined Score	# Matched Iso.	# Missed Iso.	MS Cov. [%]	Pattern Cov. [%]
396.0756	$C_{22}H_{16}O_3N^{23}NaP$	34.40	15.50	-0.96	396.07600	1	92.83	4	1	96.08	97.43

Figure S17 Found and calculated HRMS-ESI of 3-(diphenylphosphoryl)isoquinoline-1-carboxylic acid.

Eu(^{3}DPIQC)$_3$

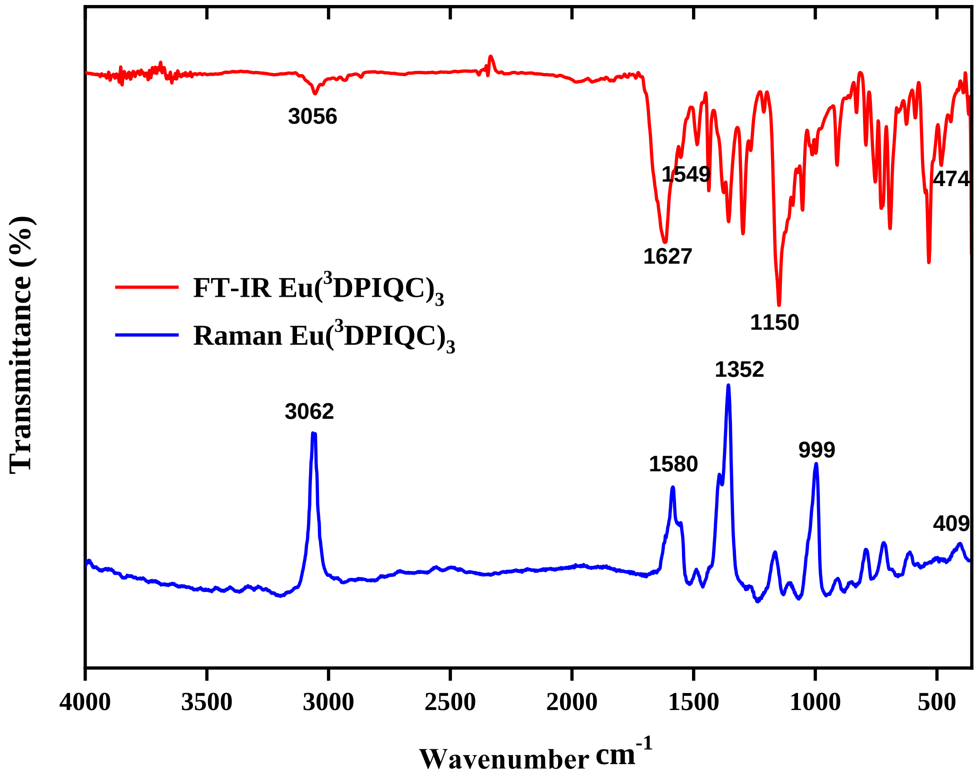

Figure 18 FT-IR and Raman spectrum of Eu(^{3}DPIQC)$_3$.

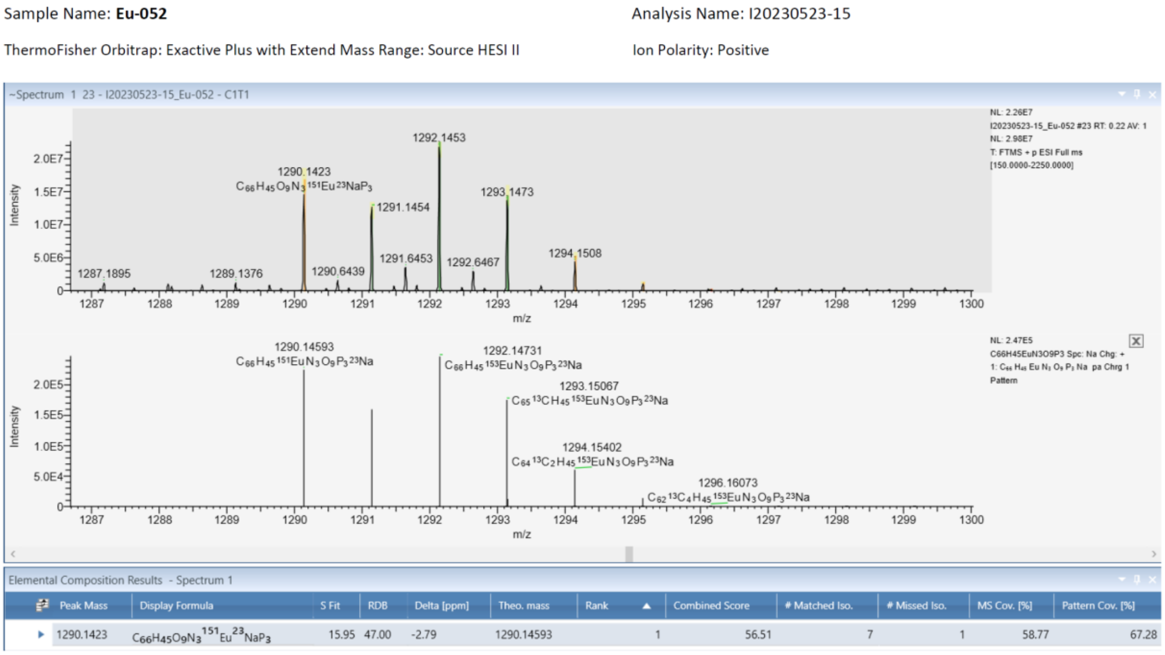

Figure S19 Found and calculated HRMS-ESI of Eu(^{3}DPIQC)$_3$.
